# Ethnopharmacological considerations of plants traditionally used by local communities to manage maternal conditions in Tanzania: a scoping review

**DOI:** 10.3389/fphar.2025.1713947

**Published:** 2026-02-17

**Authors:** Mwanaidi Omary, Maryyusta Nguyamu, Jackline Nkoma, Hamisi S. Japhari, Obadia K. Bishoge, Emanuel L. Peter

**Affiliations:** 1 Mabibo Traditional Medicine Research Centre, National Institute for Medical Research, Dar es Salaam, Tanzania; 2 Department of Environmental and Occupational Health, Muhimbili University of Health and Allied Sciences, Dar es Salaam, Tanzania

**Keywords:** ethnopharmacology, maternal conditions, maternal health, medicinal plants, Tanzania

## Abstract

**Ethnopharmacological relevance:**

Despite notable progress in maternal health and a reduction in maternal mortality rates, Tanzania still falls short of global targets. Local women increasingly use herbal medicine to manage maternal conditions, highlighting the need of documenting and evaluate these traditional practices.

**Aim of the study:**

In this study, we aimed to identify the medicinal plants commonly used by women to manage maternal conditions and to critically evaluate the available scientific evidence regarding their efficacy and safety.

**Methods:**

A scoping review was conducted in accordance with the framework of the Preferred Reporting Items for Systematic Reviews and Meta-Analyses extension for scoping reviews (PRISMA-ScR). Articles were retrieved from PubMed, Web of Science, Scopus, African Index Medicus (AIM), Maternity and Infant Care (MIC), and CINAHL, covering the period from inception to July 2025. Eligible studies were screened for relevance and taxonomic accuracy. Data were analyzed using descriptive statistics (frequency distribution and percentages) in Microsoft Excel.

**Results:**

A total of 330 plant species from 82 families were identified across 14 regions. Morogoro, Pwani, and Kagera exhibited the highest species diversity. The most represented families were as follows: Fabaceae (57 species), Rubiaceae (22 species), and Asteraceae (18 species). Frequently cited plants included *Azadirachta indica* A. Juss. (five citations), *Annona senegalensis* Pers., (four citations), and *Ricinus communis* L. (four citations). Twelve maternal conditions were reported to be managed using at least one of these plant species. However, only 23 species (7%) had scientific evidence supporting their traditional use, and only 74 species (22%) had safety data confirming non-toxicity.

**Conclusion:**

Tanzanian women utilize a wide range of medicinal plants to manage maternal conditions; however, only a small proportion of these plants have been scientifically validated or have safety data. Further pharmacological and toxicological studies are needed to verify their efficacy and ensure maternal safety. Healthcare providers should remain aware of potential concurrent herbal use during clinical encounters to ensure optimal patient care.

## 1 Introduction

Globally, maternal mortality remains a pressing concern, with nearly 800 women estimated to die each day from preventable complications linked to pregnancy and childbirth. A large proportion of these deaths occur in low- and lower-middle-income countries (WHO, 2023). Tanzania has made notable progress in reducing its maternal mortality ratio (MMR), from approximately 530 deaths per 100,000 live births in 2015/2016 to 104 per 100,000 live births in 2022 (TDHS, 2022). Although this decrease in the MMR is a significant achievement, the figure remains above the Sustainable Development Goal (SDG) target 3.1, which aims to lower maternal death to less than 70 maternal deaths per 100,000 live births by 2030 (UN, 2023). Tanzania’s success is attributable to increased political commitment, an increased number of emergency obstetric and newborn care (EmONC) facilities, a growing health workforce, a strengthened obstetric referral network, capacity building, mentorship, and the conduct of maternal and perinatal death reviews and surveillance at all levels (Africa CDC, 2025). Sustaining the current gain in MMR reduction and achieving the SDG targets requires innovative strategies tailored to the local context, such as structured integration of traditional and herbal medicines, especially in regions where such practices are culturally significant and accessible (TDHS, 2022).

Traditional medicine, especially herbal remedies, has long been used by women of reproductive age to manage pregnancy-related conditions (Japhari et al., 2025; Makombe et al., 2023). These remedies are often valued for their accessibility, affordability, and potential to alleviate symptoms such as nausea, fatigue, and stress (Mudonhi and Nunu, 2022). Nonetheless, without proper regulation, quality assurance, and integration into formal health systems, their safety and effectiveness remain uncertain. For instance, studies from Zambia and other countries in Sub-Saharan Africa have documented the common use of plants such as lemons for nausea/vomiting and the common cold, soybean to boost energy, ginger (*Zingiber officinale* Roscoe; family: Zingiberaceae) for the common cold and nausea/vomiting, and neem (*Azadirachta indica* A. Juss.; family: Meliaceae) to prevent pregnancy and as an abortifacient (Dika et al., 2017; Hajj et al., 2020; John and Shantakumari, 2015).

Persistent contributors to maternal mortality, including postpartum hemorrhage, infections, hypertensive disorders, delivery complications, and unsafe abortion, remain difficult to control through conventional measures alone (WHO, 2023). This situation thus requires exploring local, innovative solutions to address maternal conditions. Although several ethnopharmacological surveys conducted in Tanzania highlight that women use medicinal plants for managing maternal conditions, recording detailed knowledge of plant species, preparation techniques, and routes of application (Abdallah et al., 2007; Dika et al., 2017; Kessy and Msalale, 2020; Kingo and Maregesi, 2020; Millinga et al., 2022; Moshi et al., 2012; Shangali et al., 2008), scientific evidence to validate their efficacy and safety remains largely unknown (Ahmed et al., 2018). Moreover, there has been no comprehensive review mapping the pharmacological and toxicological evidence of these plants. This situation underscores the need for a systematic review of medicinal plants used for maternal health in Tanzania, along with a critical evaluation of the available pharmacological and toxicological data. The present scoping review addresses this gap by cataloging medicinal plants that are traditionally used for maternal conditions and appraising the extent to which their use is supported by scientific research. This approach provided insights into plant species, their traditional applications, and available scientific evidence validating their use, which not only informs priority plants for future research but also influences practice and policy.

## 2 Methods

### 2.1 Review procedures

In this study, we followed the framework of the Preferred Reporting Items for Systematic Reviews and Meta-Analyses extension for scoping reviews (PRISMA-ScR) (Tricco et al., 2018), ensuring transparency and reproducibility in the review process.

### 2.2 Data sources and selection criteria

Relevant articles were identified through systematic searches of the PubMed, CINAHL, Scopus, African Index Medicus (AIM), and Maternity and Infant Care (MIC) databases, covering all available publications up to July 2025. The primary search used keywords grouped into three categories; the first category included “medicinal plant (s),” “herbal medicine,” “traditional medicine (s),” and “traditional therapy”; the second category included “maternal condition (s)” and “maternal health”; and the third category included “Tanzania” and “United Republic of Tanzania.” The three search categories were combined with the Boolean logic term “AND,” whereas the keywords within each category were combined with “OR.” The secondary search paired the names of individual plants with specific maternal conditions to obtain experimental evidence and toxicity profiles. The searches were updated before the final synthesis to include the most recent studies.

Screening was carried out independently by two reviewers using the Rayyan web tool (Ouzzani et al., 2016). Two independent reviewers (MO and HS) screened titles and abstracts to identify eligible articles using the predefined criteria. The full texts of the eligible articles were obtained and assessed against the inclusion and exclusion criteria. Any disagreements between the reviewers during the full-text assessment were resolved through discussion and consensus, and when no resolution was reached, a third reviewer (ELP) was involved in the final decision. Articles were considered eligible if they (1) were ethnomedical surveys conducted in Tanzania, (2) reported plants used traditionally by Tanzanian communities, (3) reported ethical approval, (4) were published in either English or Kiswahili, because these are the official languages of the United Republic of Tanzania and it is expected that the majority of research workers in ethnopharmacology can adopt local languages to engage with traditional healers and the general community. Studies were excluded if they (1) lacked the binomial Latin name of the plants, (2) did not report the outcome of interest, or (3) were review articles without primary data.

### 2.3 Quality assessment

The scientific names of all the reported species were cross-checked with the World Flora Online database at https://wfoplantlist.org/online.org. Any discrepancies between the reported and verified names were noted and tabulated.

### 2.4 Data synthesis and reporting

Extracted data included Latin binomial names, plant family, vernacular names, voucher number, maternal conditions for which the plants were used, other reported uses, plant part(s), traditional method(s) of preparation, and geographical distribution. Where experimental evidence was available, details such as the extract type, dose, route of administration (RoA), test system (animal or human), mechanisms of action, and toxicity outcomes were captured. The extracted data were migrated to Microsoft Excel, summarized into descriptive statistics, and presented in tables, charts, and spatial mapping. Validation of traditional claims and safety was reported in thematic and tabular forms.

### 2.5 Ethnopharmacological indices

Two indices were applied to quantify ethnobotanical importance:

- Fidelity level (FL): this index measures how consistently a species is reported for a specific maternal condition relative to all its uses; FL= (Ns/FCs) × 100, where *Ns* is the number of informants citing the plant for a particular use and *FCs* is the total number of informants who mentioned the plant for any use (Andrade-Cetto and Heinrich, 2011). In this review, the authors of the retrieved articles (Ns) were regarded as informants to facilitate FL calculation.
- Relative frequency of citation (RFC): This index reflects the popularity of a species based on the proportion of informants who mention it. It is obtained by dividing the number of informants mentioning the use of species X by the total number of informants (Leonti, 2022).

## 3 Results

### 3.1 Summary of the studies included

**FIGURE 1:**
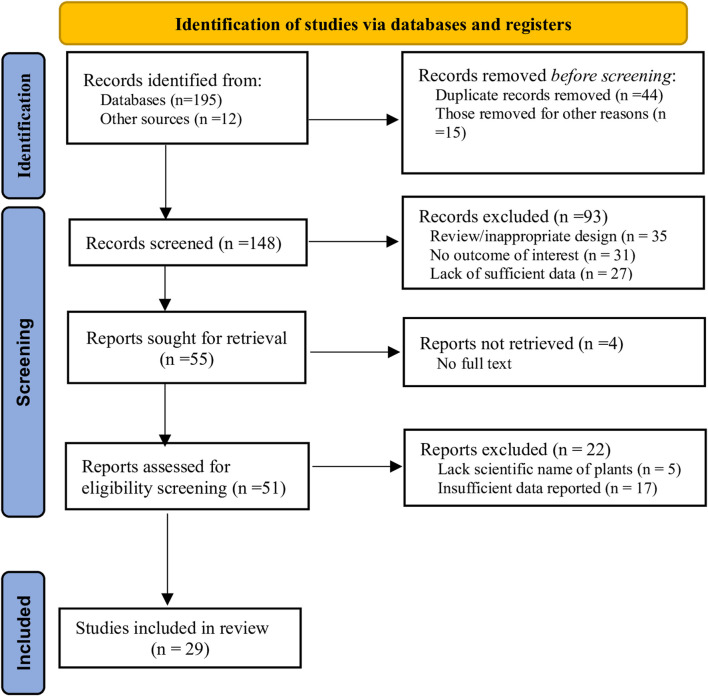
Flow diagram of the screened, included, and excluded studies.

A total of 207 articles were retrieved from databases and through a manual search. These articles were assessed for relevance and screened against the predetermined inclusion criteria. Consequently, 29 articles were finally included (Figure 1). The majority (20) of these articles were ethnomedicinal/ethnobotanical surveys conducted in different regions in Tanzania; five (05) articles were clinical trials, and four (04) were experimental studies.

### 3.2 Traditional use of plants for maternal conditions

Approximately 330 plant species from 82 families were reported for the traditional management of maternal conditions in Tanzania. Supplementary Table S1 summarizes key information that includes the scientific name of the plant species, their family, local name, voucher number, region, parts used, method of preparation (MoP), RoA, and other uses (Supplementary Table S1).

### 3.3 Distribution of medicinal plants

**FIGURE 2:**
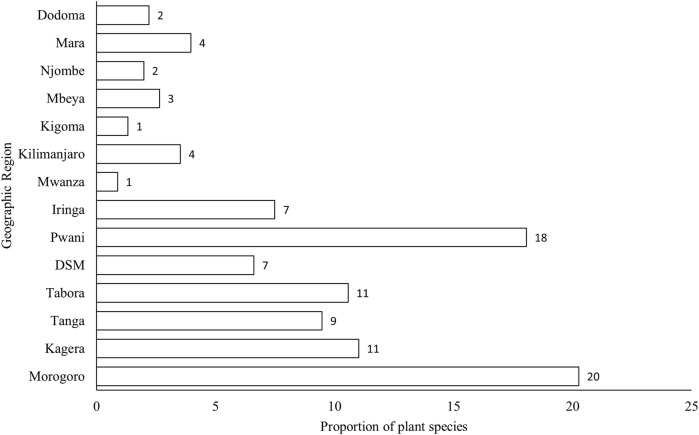
Distribution of medicinal plants in regions in Tanzania.

The recorded medicinal plants (330) were from 14 regions in Tanzania. The majority (20%) of the medicinal plants used for maternal conditions were found in the Morogoro region, whereas the lowest percentage (1%) was reported in the Mwanza region (Figure 2).

### 3.4 Diversity of medicinal plants

**FIGURE 3:**
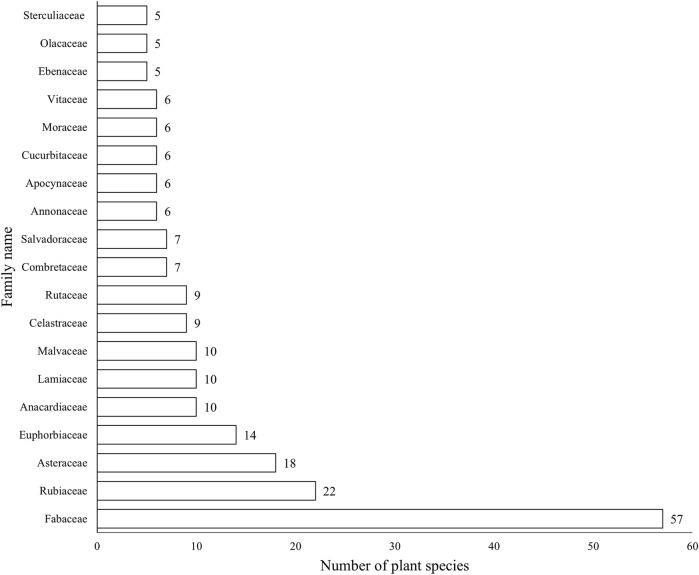
Plant families with high diversity.

A total of 82 plant families were reported by local communities in Tanzania to be used in the management of 11 maternal conditions. The top recorded families were the following: Fabaceae 17% (57 species), Rubiaceae 7% (22 species), Asteraceae 5% (18 species), Euphorbiaceae 4% (14 species), Anacardiaceae 3% (10 species), Lamiaceae 3% (10 species), and Malvaceae 3% (10 species) (Figure 3).

### 3.5 Methods of preparation (MoP), modes of application (MoA), and plant parts used (PU)

**FIGURE 4:**
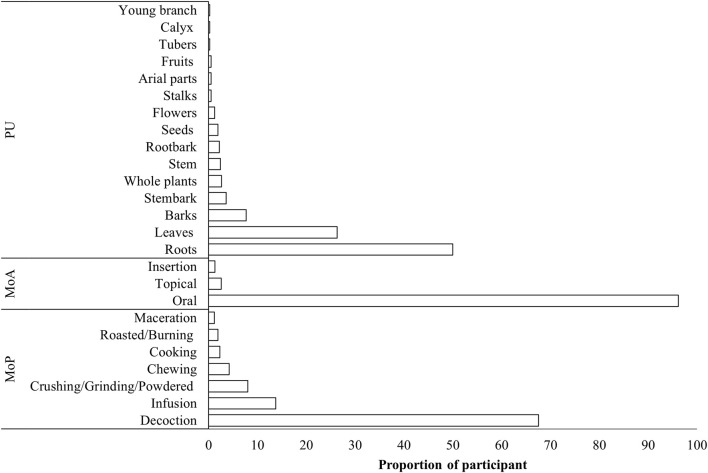
Methods of preparation, modes of application, and plant part used.

The most common MoP for the remedies was decoction (68%), followed by infusion (14%), crushing/grinding (8%), chewing (4%), cooking (2%), roasting/burning (2%), and maceration (1%). The oral route (96%) was the most common MoA, followed by topical applications (3%) and insertion of remedies into the vagina (1%). The most commonly utilized plant part was the root (50%), followed by leaves (26.3%), bark (7.7%), stem bark (3.6%), the whole plant (2.6%), the stem (2.4%), root bark (2.2%), seeds (1.9%), and flowers (1.2%). Other plant parts used included stalks, aerial parts, and fruits (0.5% each), along withtubers, calyxes, and young branches (0.2% each) (Figure 4).

### 3.6 Ethnopharmacological indices

#### 3.6.1 Fidelity level (FL)

The FL is grouped according to the maternal conditions reported. Each maternal condition was mentioned by at least two informants. According to Table 1, *A. indica* had the highest number of informants for any use and for specific use compared to the other plant species identified for abortion.

**TABLE 1:** Fidelity level grouped according to the maternal conditions reported.

Maternal condition	Plant species (family)	TIS	TIU	Fidelity level %	References
Inducing abortion	*Aloe* sp. (Asphodelaceae)	2	4	50	Nikolajsen et al. (2011), Rasch et al., 2014; Lindh (2015), Nikolajsen et al. (2011), and Rasch et al. (2014)
	*Azadirachta indica* A. Juss. (Meliaceae)	4	5	75	Dika et al., 2017; Lindh (2015), Millinga et al. (2022), Nikolajsen et al. (2011), and Rasch et al. (2014)
	*Vernonia amygdalina* Delile (Asteraceae)	3	3	100	Kingo and Maregesi (2020), Nikolajsen et al. (2011), and Rasch et al. (2014)
	*Commelina africana* L. (Commelinaceae)	2	2	100	Nikolajsen et al. (2011) and Rasch et al. (2014)
	*Zehneria scabra* (Linn. F.) Sond. (Cucurbitaceae)	2	2	100	Nikolajsen et al. (2011) and Rasch et al. (2014)
	*Cassia mimosoides* L. (Fabaceae)	2	2	100	Nikolajsen et al. (2011) and Rasch et al. (2014)
	*Desmodium barbatum* (L.) Benth (Fabaceae)	2	2	100	Nikolajsen et al. (2011) and Rasch et al. (2014)
	*Macrotyloma axillare* (E. Mey.) Verdc. (Fabaceae)	2	2	100	Nikolajsen et al. (2011) and Rasch et al. (2014)
	*Ocimum suave* Willd. (Lamiaceae)	2	3	75	Kitula (2007), Nikolajsen et al. (2011), and Rasch et al. (2014)
	*Sphaerogyne latifolia* Naudin (Melastomataceae)	2	2	100	Nikolajsen et al. (2011) and Rasch et al. (2014)
	*Crossopteryx febrifuga* (G. Don) Benth. (Rubiaceae)	3	4	100	Augustino and Gillah (2005), Chhabra et al. (1991), Hedberg et al. (1983), and Maregesi et al. (2007)
	*Oldenlandia corymbosa* L. (Rubiaceae)	2	2	100	Nikolajsen et al. (2011) and Rasch et al. (2014)
	*Canthium* sp. (Rubiaceae)	2	2	100	Nikolajsen et al. (2011) and Rasch et al. (2014)
	*Rubia cordifolia* L. (Rubiaceae)	2	2	100	Nikolajsen et al. (2011) and Rasch et al. (2014)
	*Obetia radula* (Baker) B.D. Jacks. (Urticaceae)	2	2	100	Nikolajsen et al. (2011) and Rasch et al. (2014)
Infertility	*Ozoroa mucronata* (Krauss) (Anacardiaceae)	2	2	100	Abdallah et al. (2007) and Chhabra et al. (1987)
	*Maytenus senegalensis* (Lam.) Exell (Celastraceae)	2	2	100	Augustino et al. (2011) and Chhabra et al. (1989)
	*Chenopodium ambrosioides* L (Chenopodiaceae)	2	2	100	Chhabra et al. (1989) and Maregesi et al. (2007)
	*Combretum molle* G. Don (Combretaceae)	2	3	75	Augustino and Gillah (2005) , Chhabra et al. (1989) , and Hedberg and Hedberg (1982)
	Se*negalia brevispica* (Harms) Seigler and Ebinger (*Acacia brevispica*) (Fabaceae)	2	4	50	Abdallah et al. (2007), Chhabra et al. (1989), Hilonga et al. (2019), and Maregesi et al. (2007)
	*Jasminum fluminense* Vell. (Oleaceae)	3	3	100	Hilonga et al. (2019)
	*Toddalia asiatica* (L.) Lam. (Rutaceae)	2	2	100	Kideghesho and Msuya (2010) and Shangali et al. (2008)
Menstrual problems	*Sorindeia madagascariensis* DC. (Anacardiaceae)	2	2	100	Chhabra et al. (1987) and Hedberg et al. (1982)
	*Uvaria acuminata* Oliv. (Annonaceae)	2	2	100	Chhabra et al. (1987) and Hedberg et al. (1982)
	Ehretia amoena Klotzsch (Boraginaceae)	3	4	75	Abdallah et al. (2007), Chhabra et al. (1987), and Hedberg and Hedberg (1982)
	*Elaedendron* *schweinfurthianum* Loes. (Celastraceae)	2	2	100	Chhabra et al. (1989) and Chhabra et al. (1984)
	*Vachellia tortilis* (Forssk.) Galasso and Banfi (*Acacia tortilis* (Forssk.) Hyne) (Fabaceae)	2	2	100	Chhabra et al. (1990) and Maregesi et al. (2007)
	*Harrisonia abyssinica* Oliv. (Rutaceae)	3	3	100	Hedberg et al. (1983), Lindh (2015), and Maregesi et al. (2007)
Anemia	*Hibiscus sabdariffa* L. (Malvaceae)	2	2	100	Lindh (2015) and Peter et al. (2014)

TIU, total informants for any use; TIS, total informants for a specific use.

#### 3.6.2 Relative frequency of citation (RFC)

Approximately 330 plant species were recorded in the reviewed articles. The plant species with the highest number of citations was *A. indica* (five citations), followed by *A. senegalensis*, *R. communis*, *C. cajan* (L.), *S. brevispica*, *S. longepedunculata*, *V. infausta*, *C. febrifuga*, *Z. chalybeum*, and *Aloe sp*., *with* four citations each. These plants were used for 12 maternal conditions, namely, menstruation problems, labor induction, abortion, lactation, pregnancy disorders, infertility, placenta expulsion, uterine problems, anemia, mastitis, galactagogue, and contraception. It should be noted that the reported medicinal plants treat two or more maternal conditions.

Sterility/infertility (117 species) and menstrual disorders (114 species) were the most frequently treated conditions, which highlights their high cultural salience and perceived therapeutic priority in traditional healthcare systems. Moderate numbers of plant species were associated with abortion care (66 species), labor induction (43), and galactagogue use (35), reflecting the broad reliance on herbal remedies throughout pregnancy and childbirth. Fewer species were reported for conditions such as pregnancy complications (20 species), expulsion of the placenta (12), and general uterine problems (9). Rarely reported conditions included mastitis (6 species), miscarriage (5), vaginal prolapse (3), and traditional contraceptive practices (2), suggesting either limited specialized knowledge or underreporting in these areas (Supplementary Table S1).

Medicinal plants cited include the following: *Aloe* spp. (*A. vera* and *A. lateritia*), which were widely used in the coastal and lake regions to regulate menstruation, induce abortion, and support lactation. *Bidens pilosa* appeared frequently in Lake Zone surveys as an abortifacient and menstrual inducer. *Combretum molle* was one of the most recurrent species and was used in Pwani, Morogoro, and Tabora for the treatment of infertility, excessive menstrual bleeding, and childbirth facilitation. *Combretum zeyheri* similarly addressed heavy menstrual flow and infertility in Tanga and Tabora. *Cussonia zimmermannii* was reported in Pwani for postpartum hemorrhage and labor induction, whereas *Ehretia amoena* was widely noted in coastal regions for dysmenorrhea, menorrhagia, and infertility. *Elaeodendron schlechterianum* (in Mara and Tabora) was used for infertility and menstrual pain, and *Guizotia scabra* was commonly cited as an abortifacient in Kagera and Mara. *Jatropha curcas* had a specialized role in treating mastitis in Kagera. *Kigelia africana* (in Mara and Morogoro) was used to stimulate lactation and manage heavy bleeding. *Lannea stuhlmannii/L. schweinfurthii* (in Morogoro, Pwani, and Kilimanjaro) were important remedies to treat infertility and facilitate childbirth. *Maytenus* spp. (in Pwani, Tanga, and Tabora) were widely used for infertility and dysmenorrhea. *Microglossa pyrifolia* supported postpartum care and the treatment of uterine prolapse in Pwani.

Several species of Rhus were also prominent: *Rhus natalensis* (in Pwani, Tanga, and Kigoma) was used for menstrual disorders, infertility, and pregnancy regulation, and *Rhus vulgaris* (in Kagera) was used to treat infertility and support childbirth. *Sorindeia madagascariensis* (in Pwani and Tanga) was frequently used for heavy menstrual bleeding and prolapse. Within the Asteraceae family, *Vernonia amygdalina* (in Kagera and Kigoma) served as an abortifacient, whereas *Vernonia lasiopus* (in Kilimanjaro and Tanga) was widely used for infertility, lactation, and ease of childbirth. *Vernonia usambarensis* (Kilimanjaro) was linked specifically to excessive menstrual bleeding. *Zaleya pentandra* (in Mara) was used to shorten labor, treat dysmenorrhea, and induce abortion (Supplementary Table S1).

### 3.7 Pharmacological evidence of some recorded medicinal plants

Of the 330 plant species identified, only 23 medicinal plants have scientific evidence to validate their application in treating fertility issues, pregnancies, labor induction, menstrual problems, and uterine prolapse. The evidence ranges from *in vitro* (three plant species), *in vivo* (21), and clinical trials (six). Based on the results, *R. communis* has *in vitro*, *in vivo*, and clinical trials data to support its use as a contraceptive by blocking ovulation, hence preventing nidation from occurring. *M*. *oleifera*, *Z*. *officinale*, and *Phyllanthus* sp. have *in vivo* and clinical data, whereas *P*. *nigrum* has only clinical data, and the remaining plant species have either *in vivo* or *in vitro* data or both to validate their uses. *M*. *oleifera* has anti-anemic effects in pregnant women, reduces the incidence of stunted growth, and can act as a galactagogue. Clinical trials and *in vivo* studies of *P*. *guava* revealed that the plant can treat dysmenorrhea by reducing pain intensity, whereas the leaves of *F*. *exasperata* alleviate dysmenorrhea by inhibiting oxytocin-induced uterine contractions in rats. In addition, the *in vivo* and *in vitro* studies showed that the following medicinal plants induce abortion: *D*. *cinerea*, *Aloe* sp., *R. communis*, *A. indica*, *V. amygdalina*, *B. pilosa*, *C. africana*, *O. suave*, *M. esculenta*, *O. corymbosa*, *Canthium* sp., and *Z. officinale*. Meanwhile, *C. cajan* and *C*. *abbreviata* have no abortifacient activity in pregnant rats. *In vivo* experiments on two species of genus *Phyllanthus* (*P*. *muellerianus* and *P*. *amarus*) improved fertility in women by inducing ovulation, restoring the estrous cycle, and treating polycystic ovary syndrome (Table 2).

**TABLE 2:** Pharmacological evidence of some recorded medicinal plants.

S/N	Plant name	Type of study	Extract/fractions, dose, and RoA		Pharmacological activity	Mechanism of action		References
1	*Abrus precatorius* L. (Fabaceae)	*In vivo*	Methanolic seed extract, 50 mg/kg p.o.		- Irregularity of the estrous cycle - Anti- implantation activity	- Decreased the duration of proestrus and estrus phases and increased the duration of metestrus and diestrus - Reversible disruption of the estrous cycle - Blocked ovulation in rats		Okoko et al. (2010)
			Petroleum ether and ethanol root extracts (100 mg/kg) p.o.		- Post- ovulatory activity - Anti- estrogenic activity when injected simultaneously with estradiol	Prevented nidation by up to 100% in albino rats		Agarwal and Ghatak (1970)
2	*Moringa oleifera* Lam (Moringaceae)	Clinical trial	Leaf flour substitution, 40% obtained Fe levels 22.68 ppm, p.o.		- Antianemia activity in pregnant women	Increased MCH, MCV, and MCHC. Increased hemoglobin levels		Loa et al. (2021)
			Powdered *Moringa* (PG) 500 mg, *Moringa* extract (EG) 500 mg, and iron folic acid/Fe 60 mg + 0.2 folic acid, p.o.		- Reduces the incidence of stunted growth	-		Basri et al. (2021)
		*In vivo*	Leaves and seed flour at a concentration of 100 mg per kg		- Impacted cognitive development - Positive effects on learning in Wistar rats	- Early maturation of the senses in the offspring compared to the control group - Reduced locomotion and greater exploration of new objects compared to the offspring in the control group		Dantas et al. (2024)
		Clinical trial	Up to 900 mg/day of *Moringa oleifera* leaves		Galactagogue	Increased breast milk volume in early postpartum patients		Fungtammasan and Phupong (2022)
		*In vivo*	Mixture of *Moringa* with *Sauropus androgynus* (L.) Merr., *Trigonella foenum- graecum* L., at 26.25, 52.5, and 105 mg/kg/day, p.o.		Induces galactagogue activity in lactating Wistar rats	-		Mustofa et al. (2020)
3	*Dichrostachys cinerea (L) Wight* et Arn. Subsp. Africana Brenan et Brummitt (Mimosaceae)	*In vivo*	Petroleum ether bark extract at concentrations of 3.2 mg/mL to 2 mg/mL		Facilitates labor in rats	- An increase in the contractile force and the frequency of muscle contractions		Rr et al. (2015)
4	*Salvadora persica* L. (Salvadoraceae)	*In vivo*	Methanolic extract at a dose of 800 mg/kg, administered intragastrically		No effect on the fertility of male or female mice	- No change in embryo weight - Caused a decrease in the relative weight of the ovary and an increase in uterine weight		Darmani et al. (2003)
5	*Psidium guajava* L. (Myrtaceae)	Clinical trial	Two extract doses (3 and 6 mg/day), p.o.		Treats primary dysmenorrhea in female students	- Reduced pain intensity		Doubova et al. (2007)
		*In vitro*	Aqueous leaf extract at a dose of (0.5 mg/mL–4.0 mg/mL)		- Treatment of primary dysmenorrhea - Inhibits contractions of the uterus	- Inhibited or abolished contractions produced by acetylcholine, oxytocin, bradykinin, carbachol, or potassium chloride in quiescent uterine horn preparations isolated from estrogen- dominated rats		Chiwororo and Ojewole (2009)
6	*Aloe* sp. (Asphodelaceae)	*In vivo*	Crude extracts of three species of Aloe; *A. globuligemma* <250 mg/kg IP; *A. chabaudii* 250 mg/kg–500 mg/kg IP; *A. cryptopoda* > 1,500 mg/kg IP		No abortifacient activity in any of the extracts in rats	- No expulsion or resorption of fetuses		Parry and Matambo (1992)
		*In vitro*	Ethanolic extract at concentrations in the organ bath: 0.04, 0.14, 0.44, and 1.40 mg/mL		Induces abortion in rats	Effect on the frequency of contractions		Nikolajsen et al. (2011)
7	*Ricinus communis* L. (Euphorbiaceae)	*In vitro*	Methanolic and aqueous extracts from the stem bark at doses of 1 and 100 μg mL^−1^		Contraceptive efficacy in vitro	- Affected ovarian cell functioning, steroidogenesis, the activity of LH on these processes, and affected normal ovulation and fecundity, leading to contraception		Nath et al. (2015)
			Ether extract of castor bean seed (IC50 = 284.30 ± 5.30 μg mL^−1^ r = + 0.9790)		Antifertility activity in vitro	- Inhibited the viability of cultured rat Decidual Stromal Cells (DSC), and bioassay- guided fractionation led to the separation of the active constituent, a colorless crystal		Zhang et al. (2007)
		*In vivo*	Seed extract		- Anti- implantation and abortifacient effects - Prolongs the estrus cycle of guinea pigs	​		Makonnen et al. (1999)
		Clinical observation	The seed extract was administered as a single oral dose of 2.3 g–2.5 g	Contraceptive efficacy in women volunteers	​		Das et al. (2000)	
		*In vivo*	An ether- soluble fraction of a methanol extract of seeds administered at doses of up to 1.2 g/kg and 600 mg/kg		Anti- implantation and anti- conceptive activities in adult female rats and rabbits	Action at several sites, including direct effects on the endometrial implantation site, on the oviduct, and/or disruption of the estrogen/progesterone balance		Okwuasaba et al. (1991)
		Clinical observation	Oral dose of castor oil (60 mL)		Induces labor in women patients (prospective evaluation)	-		Garry et al. (2000)
		*In vitro*	Castor oil		Induces labor	- Increase in the contractile activity of the castor oil– exposed myometrial strips		O’Sullivan et al. (2010)
		*In vivo*	Ethanolic extract at concentrations in the organ bath: 0.04, 0.14, 0.44, and 1.40 mg/mL		Induces abortion in rats	Effect on the force of uterine contractions		Nikolajsen et al. (2011)
8	*Azadirachta indica* A. Juss (Meliaceae)	*In vivo*	Seed extract was administered orally, 6 mL (in baboons) or 3 mL (monkeys) for 6 days		Termination of pregnancy	- Decline of chorionic gonadotrophin (CG) and progesterone - Decline in serum progesterone		Talwar et al. (1997b)
			0.6 mL of seed extracts, p.o.		Termination of pregnancy in rats	- Increases in the weight of mesenteric lymph nodes		Talwar et al. (1997a)
			Seed extract at a dose of 3 to mL, p.o.		Induces abortion in primates	​		Mukherjee et al. (1996)
			Ethanolic extract at conc. in the organ bath: 0.04, 0.14, 0.44, and 1.40 mg/mL		Induces abortion in rats	- Effect on the frequency of contractions		Nikolajsen et al. (2011)
9	*Hibiscus sabdariffa* L. (Malvaceae)	*In vivo*	Aqueous extract at doses of (200 mg/kg–1,000 mg/kg body wt.), p.o.		Beneficial effects on the red blood cells at low doses (200 mg–400 mg/kg) in Wistar albino rats	-		Adigun et al. (2006)
​	​	Clinical trial	Extract at doses 1,000, 1,500, and 2,000 mL/day, p.o.		Improves hematopoietic parameters in mildly anemic adults	-		Peter et al. (2017)
10	*Vernonia amygdalina* Delile (Asteraceae)	*In vitro*	Aqueous extracts (100 mg/mL–400 mg/mL)		Induces abortion	Increases in uterine smooth muscle cell contractility at the tested dose		Attah et al. (2012)
11	*Bidens pilosa* L (Asteraceae)	*In vitro*	Ethanolic extract at concentrations in the organ bath: 0.04, 0.14, 0.44, and 1.40 mg/mL		Induces abortion	Effect on the force of uterine contractions		Nikolajsen et al. (2011)
12	*Commelina africana* L (Commelinaceae)	*In vitro*	Aqueous extracts (100 mg/mL–400 mg/mL)		Induces was abortion	Increases in uterine smooth muscle cell contractility at the tested concentration range of 100 to 400 mg/mL		Attah et al. (2012)
		*In vitro*	Ethanolic extract at concentrations in the organ bath: 0.04, 0.14, 0.44, and 1.40 mg/mL		Induces abortion	- Effect on the force of contractions - Effect on the frequency of contraction		Nikolajsen et al. (2011)
13	*Manihot esculenta* Crantz (Euphorbiaceae)	*In vitro*	Ethanolic extract at concentrations in the organ bath: 0.04, 0.14, 0.44, and 1.40 mg/mL		Induces abortion	Effect on the force of uterine contractions		Nikolajsen et al. (2011)
14	*Desmodium barbatum* (L.) Benth (Fabaceae)	*In vitro*	Ethanolic extract at concentrations in the organ bath: 0.04, 0.14, 0.44, and 1.40 mg/mL		Induces abortion	- Effect on the force of uterine contractions - Effect on the frequency of uterine contractions		Nikolajsen et al. (2011)
15	*Ocimum suave* Willd (Lamiaceae) Syn of *Ocimum gratissimum* subsp. gratissimum	*In vitro*	Ethanolic extract at concentrations in the organ bath: 0.04, 0.14, 0.44, and 1.40 mg/mL		Induces abortion	- Effect on the force of uterine contractions - Effect on the frequency of uterine contractions		Nikolajsen et al. (2011)
16	*Oldenlandia corymbosa* L (Rubiaceae)	*In vitro*	Ethanolic extract at concentrations in the organ bath: 0.04, 0.14, 0.44, and 1.40 mg/mL		Induces abortion	Effects on the force of contraction		Nikolajsen et al. (2011)
17	*Canthium* sp. (Rubiaceae)	*In vitro*	Ethanolic extract at concentrations in the organ bath: 0.04, 0.14, 0.44, and 1.40 mg/mL		Induces abortion	- Effects on the frequency of uterine contractions		Nikolajsen et al. (2011)
18	*Zingiber officinale Roscoe*	*In vivo*	Aqueous extract at a high dose of 2,000 mg/kg/day		Abortifacient and subfertility effects in female mice	Disrupted the estrous cycle and blastocyst implantation without teratogenesis		ElMazoudy and Attia (2018)
	​	Clinical observation	A dose of 500 mg of dried ginger capsules twice daily		Improves breast milk volume in women during the immediate postpartum period	-		Paritakul et al. (2016)
19	*Piper nigrum sativum L*	Clinical observation	Hot and sour soup twice a day for a week		Promotes breastfeeding in postnatal mothers	-		Ekambaram (2023)
20. M	*Ficus sp*	*In vivo*	The leaves of *Ficus exasperata* at concentrations of t 1.0 × 10(- 2) mg/mL		Alleviates dysmenorrhea	Inhibited oxytocin- induced uterine contractions in rats		Bafor et al. (2011)
21	*Phyllanthus sp*	*In vivo*	*Phyllanthus muellerianus* extract at concentrations of 30, 60, and 120 mg/kg		Increases women's fertility	Restored estrous cyclicity, induced ovulation, reduced blood glucose levels and oxidative stress, improved the lipid profile and sex hormone levels, and prevented ovarian damage in PCOS rats		Ndeingang et al. (2019)
		*In vivo*	Aqueous extract of *Phyllanthus amarus* leaves at a concentration of 0.2 mg/100 g body weight		Treats infertility But also showed abortifacient effects in treated rats	Reduced s the time frame for implantation in treated rats		Iranloye et al. (2010)
22	*Cassia abbreviata*	*In vivo*	Crude extract at doses of 500 mg/kg–750 mg/kg IP		No abortifacient activity in pregnant rats	No expulsion or resorption of the fetuses		Parry and Matambo (1992)
23	*Cajanus cajan* (L.) Millsp	*In vivo*	Aqueous extract (infusion, proportion C. cajan and A. hispidum proportion 1:1.3). Doses of 0, 150, 300, and 600 mg/kg		No abortion effects in pregnant rats	- No significant change in the mean weight of the fetuses - No change in the percentage of post- implantation loss		Lemonica and Alvarenga (1994)

### 3.8 Toxicological aspects of the mentioned medicinal plants

Only 74 out of 330 plant species have been studied for toxicity. An acute toxicity study was performed for the majority of the plant species (54), followed by a sub-acute study (18), whereas sub-chronic and chronic studies were conducted for 16 and five plant species, respectively. The results revealed that 38 plant species had no toxic effects based on *in vivo* and *in vitro* studies. However, 14 plants showed signs of toxicity, including anemia, inappetence, locomotor disturbances, paresis, renal hemorrhage, decrease in body weight, and inflammation in a dose- and time-dependent manner. These plants include *C. tomentosa*, *S. persica*, *P. pinnata*, *O. insignis*, *P*. *nigrescens*, *C*. *abbreviata*, *B*. *micrantha*, *E*. *hirta*, *J*. *curcas*, *O*. *suave*, *M. obtusifolia*, and *A*. *indica* (Adu-Amoah et al., 2014; Anywar et al., 2022; Moshi et al., 2010; Nchu et al., 2011; Rajabalian et al., 2009). Additionally, four plant species showed mortality, including *Chenopodium* and *J. curcas* (Awasthy et al., 2010; Ez-Zriouli et al., 2023). Based on these results, it should be noted that the majority of medicinal plants used locally (77%) lack scientific evidence proving their safety for human use (Table 3).

**TABLE 3:** Plants with toxicity information.

No.	Plant names	Study type	Toxicological evaluations	References
1	*Cucurbita moschata* Duchesne. (Cucurbitaceae)	*In vivo*	A 13-week oral toxicity in Sprague–Dawley rats showed no mortalities at the tested dose of up to 36,000 ppm	Kleijn et al. (2024)
2	*Abrus precatorius* L (Fabaceae)	*In vivo*	Approximately three studies on acute oral toxicity tested doses ranging from 200 mg/kg to 2,000 mg/kg of the methanolic (70%) crude extract of seeds, which were nontoxic to Wistar albino rats and mice	Adedapo et al. (2007), Barve and Ojha (2013), Tabasum et al. (2019)
			The lethal dose (LD_50_) of the abrin-derived peptide was found to be 2.25 mg/kg of body weight in normal mice	Bhutia et al. (2009)
4	*Cedrella odorata* L. (Meliaceae)	*In vivo*	Acute toxicity: mild analgesia was noted at doses ranging from 625 to 5,000 mg/kg; more autonomic system effects were noted at higher doses Sub-chronic toxicity: no sign of toxicity observed at a dose of 500 mg/kg	Giordani et al. (2015)
5	*Ficus sycomorus* L. (Moraceae)	*In vivo*	Acute toxicity: decoction of leaves had an LD_50_ value of 1,553.61 mg/kg, classified as low toxicity	Ramdé-Tiendrébéogo et al. (2014)
6	*Rubus pinnatus* Willd (Rosaceae)	*In vivo*	Genotoxicity: no genotoxic or mutagenic effects at doses of 500 and 1,000 mg/kg	Tolentino et al. (2015)
7	*Moringa oleifera* (Moringaceae)	*In vivo*	Acute toxicity: The LD_50_ of the hydroethanolic leaf extract and infusion was greater than 2,000 mg/kg. The oil did not cause any skin irritation	Aliyu et al. (2021); de Barros et al. (2022), Saleem et al. (2020), Tunit et al. (2022), Asiedu-Gyekye et al. (2014), de Siqueira Patriota et al. (2021), Ajibade et al. (2013), Awodele et al. (2012)
			Reproductive toxicity: leaf and seed flour were found to be safe to pregnant women at a concentration of 100 mg/kg	Dantas et al. (2024)
9	*Senna occidentalis* L. (Caesalpiniaceae)	*In vivo*	Acute toxicity: Two studies showed that an acute dose of 5,000 mg/kg of stem extract in rats and mice resulted in no mortality or gross abnormalities.	Mugale et al. (2021), Silva et al. (2011)
10	*Piliostigma thonningii* (Schumach.) Milne-Redh (Caesalpiniaceae)	*In vivo*	Acute toxicity: The LD_50_ was above 2,000 mg/kg for the stem bark extract	Olela et al. (2020)
11	*Capparis tomentosa* Lam (Capparidaceae)	*In vivo*	Single or repeated dosages of 5, 2.5, and 0.25 g/kg of dried leaves or stems were toxic to Nubian goats. Features of toxicity included inappetence, locomotor disturbances, paresis, especially of the hind limbs, and recumbency	Ahmed et al. (1993)
			Daily oral doses of up to 5 g/kg per day of the dried leaves show signs of toxicity in goats in a time- and dose-dependent manner	Ahmed and Adam (1980)
12	*Maytenus senegalensis* (Lam) Exell (Celastraceae) syn of *Gymnosporia senegalensis*	Clinical trial	No toxic effects at a dose of 800 mg every 8 h a day for 4 days in healthy men	Kassimu et al. (2022)
		*In vivo*	Acute toxicity: ethanolic root extract was non-toxic, and the oral median lethal dose in mice was >1,600 mg/kg	Malebo et al. (2015)
13	*Chenopodium ambrosioides* L (Chenopodiaceae)	*In vivo*	Acute toxicity: death and other clinical signs of toxicity at doses of 300, 1,000, and 2,000 mg/kg in rats (LD_50_ was 500 mg/kg, hence toxic at high doses)	Ez-Zriouli et al. (2023)
			Acute toxicity: no sign of toxicity in acute toxicity study at doses of up to 3,000 mg/kg of aqueous leaf extract in rats	da Silva et al. (2014)
			Sub-chronic toxicity: leaf extract in mice did not result in death or alterations of body weight at doses of 500 mg/kg by gavage	Pereira et al. (2010)
14	*Combretum molle* G. Don (Combretaceae)	*In vivo*	Chronic toxicity: oral aqueous extract did not result in mortality or visible signs of toxicity at a dose of 250 mg/kg	Miaffo et al. (2020)
15	*Dichrostachys cinerea (L) Wight* et Arn. Subsp. *Africana Brenan* et Brummitt (Mimosaceae)	*In vivo*	Acute toxicity: the methanolic extract did not result in mortality up to a dose of 3,500 mg/kg in mice and rats	Babu et al. (2011)
16	*Entada abyssinica* Steud. Ex A. Rich (Mimosaceae)	*In vivo*	Sub-acute toxicity: methanolic stem bark extract at 600 mg/kg was well-tolerated	Obakiro et al. (2021)
			Acute toxicity: the extract did not show any toxicity up to 2,000 mg/kg, but above this dose, the mice exhibited an increased respiratory rate and scruffy hair	Haule et al. (2012)
17	*Cajanus cajan* (L.) Millsp. (Papilionaceae)	*In vivo*	Acute toxicity: leaf extract did not result in mortality, and noted alterations in weight and behavioral abnormalities were observed at oral doses (15.0 g/kg and 11.3 g/kg) Sub-chronic toxicity: no mortality or significant variances in hematological and biochemical parameters or organ histopathology were observed at doses of 1.5, 3.0, and 6.0 g/kg	Tang et al. (2017)
		*In vivo*	Chronic toxicity: ethanolic root extract to male and female Wistar rats showed no sign of toxicity when administered at doses of 0.2 or 1.0 g/kg	Vo et al. (2023)
​	​		Acute toxicity: aqueous extract was safe at a dose of 2,000 mg/kg	Legba et al. (2019)
18	*Indigofera arrecta* A. Rich (Papilionaceae)	*In vivo*	Acute toxicity: The extract at a dose of 10 g/kg did not result in mortality Sub-chronic toxicity: a dose of 2 g/kg administered orally daily for 30 days showed no physiological change	Nyarko et al. (1999)
19	*Clausena anisata* (Willd) Benth (Rutaceae)	*In vitro*	Cytotoxicity: the extract was the least toxic with an LC_50_ of 0.17 mg/mL	Adamu et al. (2013)
20	*Zanthoxylum chalybeum* Engl. (Rutaceae)	*In vivo*	Aqueous and organic extracts were toxic to brine shrimp (LD_50_ < 1,000 μg/mL)	Musila et al. (2013)
			The ethanolic extract was nontoxic with an LC_50_ value of 38.51 in a brine shrimp lethality assay	Mbunde et al. (2017)
21	*Salvadora persica* L. (Salvadoraceae)	*In vitro*	Cytotoxicity: persica mouthwashes are toxic to macrophages, epithelial cells, fibroblasts, and osteoblasts in a concentration-dependent manner	Rajabalian et al. (2009)
22	*Dodonaea viscosa* (L.) Jacq. (Salvadoraceae)	*In vivo*	Acute toxicity: the extract did not show any overt sign of toxicity at a dose of 2,000 mg/kg Sub-acute toxicity: no signs of toxicity at a dose of 1,000 mg/kg	Teshome et al. (2010)
			Acute toxicity: the extract did not show any sign of toxicity in mice at doses of up to 5,000 mg/kg	Khalil et al. (2006)
23	*Paullinia pinnata* L. (Salvadoraceae)	*In vivo*	Acute toxicity: mice did not show any form of morbidity or mortality at a dose of 10,000 mg/kg of leaf extract Sub-acute: the extract was toxic at doses higher than 200 mg/kg. The toxicity effect was dose-dependent	Adeyemo-Salami and Makinde (2013)
24	*Ozoroa insignis* Del. Susbsp. Reticulata (Bak.f) Gillet (Anacardiacea)	*In vivo*	The ethanolic extract was toxic to mice at doses higher than 1,000 mg/kg body weight in an acute toxicity study The brine shrimp test results also showed the same pattern of toxicity as in acute, with LC_50_ = 10.63 μg/mL	Haule et al. (2012)
25	*Annona senegalensis* Pers. subsp. senegalensis. (Annonaceae)	*In vivo*	Acute toxicity: the root bark extract exhibited no toxic effects at 400 mg/kg	Okoye et al. (2012)
		*In vitro*	Total oil and its fractions showed mild to moderate cytotoxicity in a brine shrimp lethality bioassay with LC_50_ = 27.3 μg/mL	Ahmed et al. (2010)
26	*Parquetina nigrescens* (Afz.) Bullock. (Asclepiadaceae)	*In vivo*	A methanol leaf and aerial part extract at doses of 100 and 300 mg/kg showed renal hemorrhage and inflammation, and hepatic inflammation in a sub-chronic toxicity study	Adu-Amoah et al. (2014)
27	*Kigelia africana* (Lam.) Benth (Bignoniacea)	*In vivo*	The extract was nontoxic, with an LC_50_ value of 424 μg/mL for DCM and 557.92 μg/mL for the ethanol extract	Mbunde et al. (2017)
28	*Adansonia digitata* L. (Bombacaceae)	*In vivo*	Aqueous and organic extracts of the stem bark were non-toxic to brine shrimp (LD_50_ > 1,000 μg/mL)	Musila et al. (2013)
			In an acute toxicity study, no mortalities were observed up to a dose of 2,000 mg/kg of the fruit extract	Hanafy et al. (2016)
			No acute oral toxicity was observed, and the extracts were considered to be safe at a dose of 3,000 mg/kg	Mumtaz et al. (2017)
29	*Cassia abbreviata* Oliv.subsp. beareana (Holmes) Brenan. (Caesalpiniaceae)	*In vivo*	Root extract exhibited high toxicity with LC_50_ values below 12.7 μg/mL in a brine shrimp toxicity test	Moshi et al. (2006b)
30	*Tamarindus indica* L. (Caesalpiniaceae)	*In vivo*	Chronic toxicity: pulp extract was well-tolerated at the tested dose of 1,000 mg/kg daily for 6 months	Iskandar et al. (2017)
			No evidence of clinical signs in rats at a dose of 2,000 mg/mL in acute oral toxicity	Escalona-Arranz et al. (2016)
			In an acute toxicity study, the extract was found to be safe up to 2,000 mg/kg orally	Rai et al. (2018)
			In a brine shrimp toxicity study, the stem bark extract had an LC_50_ of 516.4 μg/mL, which was considered to be weakly toxic	Nguta and Mbaria (2013)
31	*Maytenus heterophylla* (E&l. & Zeyh.) N. Robs. (Celastraceae)	*In vivo*	Leaf extracts at a dose of 1,200 mg/kg were shown to be non-toxic in an acute toxicity study	da Silva et al. (2011)
32	*Combretum cfr. molle* R. Br. ex D. Don. (Combretaceae)	*In vivo*	No mortality or visible signs of toxicity at doses of the aqueous extract of 62.5, 125, and 250 mg/kg for 6 months	Miaffo et al. (2020)
33	*Crassocephalum vitellinum* (Benth.) S. Moore. (Compositae)	*In vivo*	The extract was non-toxic to mice up to 5,000 mg/kg in an acute toxicity study	Moshi et al. (2014)
34	*Dissotis rotundifolia* (Melastomataceae)	*In vivo*	The LD_50_ in mice was above 500 mg/kg	Abere et al. (2010)
35	*Psidium guajava* L. (Myrtaceae)	*In vivo*	Acute toxicity: no mortality or signs of toxicity were recorded at 5,000 mg/kg Sub-acute toxicity: significant variations in body weight, relative weight of organs, and biochemical parameters were observed at doses of 250, 500, and 1,000 mg/kg	Manekeng et al. (2019)
			The essential oil from the stem bark was toxic, with an LC_50_ value of 1.0009 (µg/mL) in a brine shrimp lethality test	Fasola et al. (2011)
36	*Ricinus communis* L. (Euphorbiaceae)	*In vivo*	Acute toxicity: the aqueous and methanol extracts did not produce any toxic signs or mortality at a dose of 2,000 mg/kg in rats Sub-chronic toxicity: no adverse effects at a dose of 1,000 mg/kg	Ilavarasan et al. (2011)
		*In vitro*	Cytotoxicity: the hydroethanolic extracts showed low toxicity (IC50 > 500 μg/mL, 24 h) against HepG2 cells	Caballero-Gallardo et al. (2023)
37	*Azadirachta indica* A. Juss (Meliaceae)	*In vivo*	Genotoxicity and maternal–fetal safety experiment; the dried leaf extract at doses of up to 1,200 mg/kg did not induce maternal toxicity, and it was neither embryotoxic nor fetotoxic	Ramalho et al. (2023)
		*In vivo*	Acute toxicity: the LD_50_ value of neem oil was 31.95 g/kg by the oral route, which is nontoxic	Deng et al. (2013)
		Clinical trials	No sign of toxicity to any of the subjects treated with 250 mL of the extract daily (morning and evening) for over 3 months	Goni Hamadama et al. (2021)
		*In vivo*	No mortality in mice treated with neem oil for 90 days at doses of 177, 533, or 1,600 mg/kg/day	Wang et al. (2013)
			Acute toxicity: the stem bark extract produced toxicity at high doses of >800 mg/kg	Mbaya et al. (2010)
38	*Hibiscus sabdariffa* L. (Malvaceae)	*In vivo*	LD_50_ above 5,000 mg/kg for the aqueous or alcoholic calyces extract	Fakeye (2008), Njinga et al. (2020), Sireeratawong et al. (2013)
39	*Zaleya pentandra* (L) Jeffrey (Aizoaceae)	*In vivo*	No toxic effects were noted for doses of up to 300 mg/kg in experimental chicks	Saleem et al. (2019)
40	*Euphorbia tirucalli* L (Euphorbiaceae)	*In vivo*	No maternal toxicity or deaths were observed after treatment with latex at a concentration of 0.05%	Silva et al. (2007)
41	*Ageratum conyzoides* (L.) L (Asteraceae)	*In vivo*	Acute toxicity: LD_50_ was 2,000 mg/kg for the aqueous extract	Palmer et al. (2019), Moura et al. (2005), Diallo et al. (2014)
42	*Canna indica* L. (Cannaceae)	*In vivo*	The single oral administration of the extract at a dose of 300 mg/kg did not cause any abnormal behavior in rats	Chigurupati et al. (2021)
43	*Bridelia micrantha* (Hochst.) Baill (Euphorbiaceae)	*In vivo*	The extract was moderately cytotoxic with a CC_50_ of 96.7 μg/mL	Anywar et al. (2022)
			The ethanol extract was categorized as mildly toxic (LC_50_ 32.0 μg/mL)	Moshi et al. (2010)
44	*Euphorbia hirta* L (Euphorbiaceae)	*In vivo*	Acute toxicity: the LD_50_ of the extract was above 5,000 mg/kg	Yuet Ping et al. (2013)
			Acute toxicity: The methanol extract of leaves exhibited mild toxic effects in mice at a dose of 5,000 mg/kg	Rajeh et al. (2012)
45	*Jatropha curcas* L. (Euphorbiaceae)	*In vivo*	Acute toxicity: the phorbol ester showed varied degrees of toxic reactions in a dose-dependent manner; 21.26 mg/kg–36 mg/kg	Li et al. (2010)
			The seed extract at 0.05, 0.5, and 1 g/kg/day showed signs of toxicity in Nubian goats and sheep	Ahmed and Adam (1979)
		*In vivo*	No clinical and biochemical signs of toxicity were observed when the leaf extract was administered at 2,000 mg/kg for 21 days	Igbinosa et al. (2013)
			The seed extract showed mortality in a time- and dose-dependent manner (1 mg/kg–30 mg/kg)	Abdu-Aguye et al. (1986)
			50% protein supplement level of *J. curcas* seeds caused biochemical alterations and mortality in rats during a feeding trial. However, all rats fed 25% survived and were healthy until the end of the experiment	Awasthy et al. (2010)
46	*Vernonia amygdalina* Delile (Asteraceae)	*In vivo*	The LD_50_ of the aqueous and ethanolic extracts was greater than 2,000 mg/kg	Dougnon et al. (2021), Legba et al. (2019)
47	*Bidens pilosa* L (Asteraceae)	*In vivo*	No signs of toxicity in mice after being supplemented with 10% conc. in food	Liang et al. (2020)
			Acute toxicity: aqueous extract had an LD_50_ greater than 5,000 mg/kg	Tcheutchoua et al. (2022)
48	*Manihot esculenta* Crantz (Euphorbiaceae)	*In vivo*	Acute toxicity: the LD_50_ was greater than 2,000 mg/kg	Dougnon et al. (2021)
49	*Ocimum suave* Willd (Lamiaceae) Syn of *Ocimum gratissimum* subsp. gratissimum	*In vivo*	Acute toxicity: the LD_50_ was up to 8,000 mg/kg	Tan et al. (2008)
50	*Rubia cordifolia* L (Rubiaceae)	*In vivo*	Acute toxicity: The LD_50_ of the crude fruit extract was greater than 1,000 mg/kg	Anantharaman et al. (2016)
51	*Rhus vulgaris* Meikle (Anacardiaceae)	*In vivo*	Acute toxicity: the extract had an LD_50_ greater than 2,000 mg/kg	Mutuku et al. (2020)
52	*Zingiber officinale* Roscoe (Zingiberaceae)	*In vivo*	Acute toxicity: no deaths occurred when the aqueous and methanolic root extracts were administered orally to mice in doses of up to 5 g/kg	Shalaby and Hamowieh (2010)
			No evidence of toxicity or death in acute and sub-acute toxicity tests in rats at maximum tolerated doses (MTDs) of 5,000 and 2,000 mg/kg body weight, respectively Chronic toxicity tests revealed an MTD and a no-observed-adverse-effect level (NOAEL) of 1,000 mg/kg body weight	Plengsuriyakarn and Na-Bangchang (2020)
			Ginger oil was not toxic to male or female rats following sub-chronic oral administration of up to 500 mg/kg per day	Jeena et al. (2011)
53	*Allium cepa* L (Amaryllidaceae)	*In vivo*	Acute toxicity: onion coat colorant (OC) at doses of 2,500, 5,000, 7,500, and 10,000 mg/kg did not result in mortality Sub-acute toxicity: doses of 5%, 2.5%, 1.25%, 0.6%, and 0.3% was safe	Kojima et al. (1993)
54	*Piper nigrum* L (Piperaceae)	*In vivo*	An acute and sub-chronic toxicity study showed that the polyherbal drug (a mixture of the seeds of *Piper nigrum* L., the leaves of *Murraya koenigii* L. Spreng., the cloves of *Allium sativum* L., and the fruits of *Garcinia quaesita* Pierre) did not cause any signs of toxicity at oral doses of 0.25 mg/kg–2.0 g/kg and (0.5, 1.0, and 1.5 g/kg), respectively, in healthy rats	Liyanagamage et al. (2020)
55	*Cucurbita pepo* L (Curcurbitaceae)	*In vivo*	The ethanolic extracts of polyherbal drug comprised of *Cucurbita pepo* L *Emblica officinalis*, *Triticum aestivum*, *Fagonia cretica*, *Momordica charantia*, and *Tribulus terrestris* revealed no treatment-related toxic manifestations or mortality. The LD_50_ was found to be >5,000 mg/kg	Ghayas et al. (2022)
56	*Arachis hypogaea* L. (Fabaceae)	*In vivo*	The ethanolic extracts of the tegument and seeds were safe in *in vitro* cytotoxic and *in vivo* genotoxicity studies up to a concentration of 2,000 mg/kg for the tegument extract and 250 mg/kg for the seed extract	Menis Candela et al. (2020)
			Leaf hydroalcoholic extracts at doses of 100, 300, or 1,000 mg/kg did not induce toxicity after repeated exposure for 28 days in rats	Cossetin et al. (2020)
57	*Citrus limon* L. (Rutaceae)	*In vivo*	Lemon extract was safe for all animal species up to the maximum proposed use levels of 1,000 mg/kg of complete feed and 250 mg/kg of drinking water	Bampidis et al. (2021)
58	*Allium sativum* L. (Amaryllidaceae)	*In vivo*	An acute and sub-chronic toxicity study showed that the polyherbal drug (a mixture of cloves of *Allium sativum* L., seeds of *Piper nigrum* L., leaves of *Murraya koenigii* L. Spreng., and fruits of *Garcinia quaesita* Pierre) did not cause any sign of toxicity at oral doses of 0.25 mg/kg–2.0 g/kg (0.5, 1.0, and 1.5 g/kg), respectively, in healthy rats	Liyanagamage et al. (2020)
59	*Cymbopogon citratus* Stapf (Poaceae)	*In vivo*	No sign of acute and sub-acute toxicity from the ethanolic leaf extracts at doses of 5,000 mg/kg b.w. and up to 1,200 mg/kg, respectively	Ayembilla et al. (2023)
		*In vivo*	Acute toxicity: the aqueous extract of the 50:50 mixture of *B. pilosa* and *C. citratus* aerial parts at doses of 2,000 and 5,000 mg/kg did not induce any apparent sign of toxicity Sub-chronic toxicity: the aqueous extract of the mixture at doses of 200, 400, and 800 mg/kg did not cause any injury to the liver, kidneys, lungs, or spleen	Tcheutchoua et al. (2022)
60	*Aloe vera* L (Liliaceae)	Clinical trial	In a pilot randomized positive-controlled trial, aloe syrup was safe and well-tolerated at a dose of 10 mL/d	Panahi et al. (2015)
		*In vivo*	No acute and sub-acute toxicity effects of Aloe syrup at a maximum concentration of 3,330 mg/kg body weight in rats. The LD_50_ was higher than 15,000 mg/kg body weight in this acute toxicity study	Wu et al. (2021)
			Methanolic flower extracts revealed no apparent signs of toxicity, nor did they result in death in albino rats in an acute toxicity study at doses of 200 mg/kg, 2, 4, 8, and 10 g/kg	Elkomy et al. (2023)
			No death or apparent behavioral changes in acute and sub-acute oral toxicity tests at doses of up to 5,000 mg/kg and 800 mg/kg, respectively	Nalimu et al. (2022)
			Hydroalcoholic leaf extracts were safe in acute and sub-acute toxicity studies at doses of up to 2,560 mg/kg when administered to chicks	Nghonjuyi et al. (2016)
61	*Securidaca longipedunculata* Fresen. (Polygalaceae)	*In vivo*	Acute toxicity: The extract at 2,000 mg/kg did not show any signs of toxicity in mice	Kola et al. (2023)
62	*Toddalia asiatica* (L) Lam. (Rutaceae)	*In vivo*	Aqueous, methanol, ethyl acetate, and n-hexane extracts from the leaves, roots, and fruits did not show acute toxicity at the highest tested concentration of 1,000 mg/kg in mice	Orwa et al. (2013)
63	*Aloe lateritia* (Asphodelaceae)	*In vivo*	The whole plant extract was highly toxic to brine shrimp, with an LC_50_ value of 19.1	Moshi et al. (2006b)
64	*Diospyros fischeri* Gurke (Ebenaceae)	*In vivo*	The root extract exhibited low toxicity to brine shrimp, with LC_50_ values between 45.4 and 95.4 μg/mL at doses between 100 and 1,600 mg/kg	Moshi et al. (2006a)
65	*Euclea divinorum* Hiern (Ebenaceae)	*In vivo*	Aqueous and ethanolic root extracts were also found to be safe at 2,000 mg/kg in an acute toxicity study in Sprague–Dawley rats	Woldemedhin et al. (2017)
66	*Grewia bicolor* Juss (Malvaceae)	*In vitro*	Cytotoxicity results showed that the extract was less toxic to HeLa cells at concentrations of up to 35 mg/mL	Masisi et al. (2021)
67	*Hymenocardia acida* Tul (Phyllanthaceae)	*In vivo*	An acute toxicity test showed that the extract was slightly toxic, with an estimated median lethal dose of 1,767.77 mg/kg body weight	Obidike et al. (2011)
			Ethanolic stem bark was toxic to brine shrimp and caused chromosomal damage in rat lymphocytes, with an LD_50_ value of 24.12(µg/mL)	Sowemimo et al. (2007)
68	*Margaritaria discoidea* (Baill.) G.L. Webster (Phyllanthaceae)	*In vivo*	The methylene chloride extract of the leaves did not cause any acute toxicity in mice at a dose of 5 × IC_50_	Cho-Ngwa et al. (2010)
69	*Markhamia obtusifolia* (Bak.) Sprague (Bignoniaceae)	*Invitro*	Cytotoxicity: the lethal concentration (LC_50_) for the aqueous extract was 0.476 mg/mL, which was relatively high (low toxicity) compared to the highly toxic berberine LC_50_ of 9.80 μg/mL	Nchu et al. (2011)
70	*Parinari curatellifolia* Benth (Chrysobalanaceae)	*In vivo*	Sub-acute toxicity: the methanol stem bark extract was slightly toxic to the liver Acute toxicity: the LD_50_ was found to be greater than 5,000 mg/kg	Emmanuel et al. (2023)
71	*Pennisetum purpureum* Schumach. (Poaceae)	*In vivo*	The extracts were non-cytotoxic up to a test dose of 100 μg/mL Acute toxicity: A dose of 2,000 mg/kg showed no sign of toxicity in mice	Ezeani et al. (2022)
72	*Ziziphus mucronata* Willd (Rhamnaceae)	*In vivo*	The methanolic bark extract was shown to be non-toxic in a brine shrimp bioassay with 8.33% and 15.18% mortality rates after 24 h and 48 h, respectively	Khumalo et al. (2021)
73	*Balanites aegyptiaca* (L.) Delile (Balanitaceae)	*In vivo*	The aqueous bark extract did not show any signs of toxicity when administered orally up to 2,000 mg/kg, but rats died when injected intraperitoneally with doses of 1,000 mg/kg	Mohamed et al. (1999)
74	*Cocos nucifera* L. (Arecaceae)	*In vivo*	Acute and sub-acute toxicity study: no mortality or clinical signs of toxicity in ethyl acetate-soluble proanthocyanidins of the immature inflorescence at the dose of 2,000 mg/kg body weight in acute and 1.75, 3.5, 7, and 14 mg/kg body weight in sub-acute toxicity studies	Ekanayake et al. (2019)
			Fermented virgin coconut oil was safe in treated rats at a dose of 5,000 mg/kg in acute, sub-chronic, and chronic studies	Ibrahim et al. (2016)
75	*Momordica charantia* L. (Cucurbitaceae)	*In vivo*	The extract of dry leaves presents cytotoxicity and low maternal toxicity at concentrations of 500, 1,000, or 2,000 mg/kg	Trautenmuller et al. (2023)
			In acute toxicity and sub-acute toxicity studies, the seed extract revealed no mortality, morbidity, or abnormal pathological or biochemical alterations in Wistar rats at doses of up to 1,000 mg/kg	Chung et al. (2022)

## 4 Discussion

The review shows that women in Tanzania rely on a wide variety of plants for maternal care. These findings highlight both the country’s rich biodiversity and the central role that traditional medicine plays in reproductive health. However, the concentration of plant species in only a subset of regions suggests that significant knowledge remains undocumented, emphasizing the importance of continued ethnobotanical surveys and the need for a national medicinal plant registry.

The predominance of Fabaceae, Asteraceae, and Rubiaceae as leading plant families for maternal remedies aligns with studies conducted in the Philippines, Nigeria, Cameroon, and Ethiopia, where these families also dominated pregnancy-related uses (Asmare et al., 2018; Magtalas et al., 2023; Ogunlakina and Sonibare, 2020; Tsobou et al., 2016). The predominance of Fabaceae and Asteraceae in maternal conditions and contraception was also reported in another study in Uganda (Adia et al., 2025). The observed similarities in diverse ecological zones indicate that these plant families are both extensively distributed and culturally accepted.

Preparation methods were largely based on decoctions, which is likely due to their simplicity and low cost. Furthermore, oral administration was the overwhelmingly preferred route. These preparations and administration patterns were also documented in other African and Asian countries (Adamolekun et al., 2023; Ahmed et al., 2018; Asmare et al., 2018; Magtalas et al., 2023; Mashile et al., 2019). Roots were the most harvested plant part, which raises concerns about ecological sustainability and the potential threat of overexploitation. This calls for complementary strategies, such as cultivating medicinal species or promoting the use of aerial parts where possible (Ahmed et al., 2018; Asmare et al., 2018; Magtalas et al., 2023).

The most frequently mentioned medicinal plants used for labor induction or abortion were *C. febrifuga*, *Aloe* sp., *R. communis*, *A. indica*, and *F. thonningii*. These frequently mentioned plants are more specific in their use and, therefore, could be given higher priority for pharmacological studies. Consistent with the present study, a systematic and scoping review conducted in an African context reported that *R. communis* and *Aloe* were used to aid labor (Adamolekun et al., 2023; Ahmed et al., 2018; El Hajj and Holst, 2020). *R. communis* was mentioned to be used as a contraceptive. In addition, Tanzanian women used *C. cajan* and *A. senegalensis* for pregnancy-related disorders such as abdominal pain, fever, and nausea. A review from Ethiopia reported that *Aloe* is used to retain the placenta and to treat breast infections (Asmare et al., 2018). The variation in reported uses of these plants may be due to their locations, variation in chemical composition, historical context, cultural practices, and traditional knowledge.

our review demonstrates that Tanzanian traditional knowledge systems harbor a substantial yet uneven repertoire of medicinal plants used to manage maternal health conditions. While the high diversity of species associated with infertility, menstrual disorders, and peri-partum care underscores the cultural centrality of reproductive health, it also exposes critical gaps in mechanistic understanding, pharmacological validation, and safety profiling. The limited number of species reported for conditions such as miscarriage, postpartum complications, and contraception further highlights areas where ethnomedical knowledge may be fragmented, restricted, or undergoing erosion.

Addressing these disparities requires a shift from descriptive ethnobotany to an integrated, evidence-driven research agenda. Priority should be given to botanical authentication, phytochemical characterization, toxicological assessment, and mechanistic studies capable of linking traditional indications to biological pathways. Equally important is the need for culturally grounded qualitative research to elucidate knowledge transmission patterns, healer specialization, and sociocultural constraints surrounding sensitive reproductive conditions.

Among plants with toxicological data, the majority have only preliminary safety evaluations, which are typically limited to acute and sub-acute toxicity assays. These studies are relevant as they provide foundational data on short-term tolerability, identify early organ-specific toxicities, and inform safe starting doses for more advanced investigations, in line with OECD and WHO guidelines (EMA, 2018). However, reliance on these assays alone presents substantial limitations: they do not capture long-term, cumulative, reproductive, genotoxic, or carcinogenic risks, nor do they assess herb–drug interactions, which are critical in populations with high polypharmacy (Shaw et al., 2012). Consequently, although acute and sub-acute assays are necessary preliminary steps, they provide an incomplete toxicological profile, underscoring the need for comprehensive sub-chronic, chronic, and mechanistic studies before the broad therapeutic or commercial use of herbal products.Pharmacological evidence highlights the effects of the reported medicinal plants on reproductive physiology, underscoring the need for contextualized and dose-specific interpretations. Medicinal plant species, including *Abrus precatorius*, *Ricinus communis*, and *Azadirachta indica*, demonstrate robust antifertility, anti-implantation, and uterotonic activities, thus supporting traditional claims but also raising safety concerns for women of reproductive age. At the same time, plants such as *Phyllanthus muellerianus*, *Psidium guajava*, and *Ficus exasperata* exhibit fertility-supportive or anti-dysmenorrheic properties, suggesting therapeutic value when appropriately applied.

The consistent galactagogue effects of *Moringa oleifera*, *Zingiber officinale*, and *Piper nigrum*, validated in clinical and experimental models, underscore their potential integration into maternal health interventions. Additionally, the hematopoietic benefits observed for *Moringa oleifera* and *Hibiscus sabdariffa* further support their utility in managing anemia, which is a critical public health challenge in Tanzania and many low-resource settings. Importantly, plants traditionally regarded as abortifacients, such as *Cassia abbreviata*, *Cajanus cajan*, and *Aloe species*, showed no such activity, revealing the limitations of unverified ethnomedical assumptions. Overall, these findings emphasize the dual need to harness the promising therapeutic properties of these plants while strengthening the regulatory, toxicological, and clinical frameworks to ensure the safe use of reproductive-active botanicals.

By identifying both the richness and the limitations of current traditional practices, in this work, we provide a foundation for strategic prioritization of species with the highest potential for therapeutic advancement. Plant species with high FL/RFC values, such as *Azadirachta indica* and *Ricinus communis,* could be prioritized for preclinical and clinical studies. Bridging ethnomedical knowledge with modern biomedical science is essential not only for developing safe, effective, and contextually appropriate maternal healthcare interventions but also for safeguarding cultural heritage and informing national and regional health policy. These findings underscore the urgent need for interdisciplinary collaboration to translate traditional botanical resources into validated, scalable, and equitable solutions for maternal health. However, this review did not include studies from all administrative regions in Tanzania and considered only articles published in Kiswahili or English, which could have understated the actual number of plant species used by local Tanzanian communities for maternal conditions. Thus, the results should be interpreted in light of these limitations.

## 5 Conclusion

Tanzanian women utilize a wide range of medicinal plants to manage maternal conditions, yet only a small proportion of these plants have been scientifically validated or have safety data. Further pharmacological and toxicological studies are needed to verify their efficacy and ensure maternal safety. Healthcare providers should remain aware of the potential of concurrent herbal use during clinical encounters to ensure optimal patient care. Finally, conservation strategies could be strengthened for the identified root-harvested plant species.
