# Supplementary material for: Ethnopharmacological considerations of plants traditionally used by local communities to manage maternal conditions in Tanzania: a scoping review
Source: Front Pharmacol. 2026 Feb 17;16:1713947. doi: 10.3389/fphar.2025.1713947 (PMC12953572; doi:10.3389/fphar.2025.1713947)
Supplement: Supplementary file 1 [file Table1.docx]

**Table 1: List of Medicinal plants, including their family, local name, Voucher no, region, parts used, mode of preparation (MoP), route of application (RoA), and other uses**

| **Family** | **Scientific name** | **Local name** | **Voucher #** | **Region** | **Parts used** | **Traditional Use** | **MoP and RoA** | **Other uses** | **References** |
| --- | --- | --- | --- | --- | --- | --- | --- | --- | --- |
| Acanthaceae | *Thunbergia alata* Boj. ex Sims | Nakagwinda, Gisanyurwa (Kinyarwanda) | 2920 | Morogoro, Kagera | Leaves | Against menorrhagia, Oxytocic action, Used for the relief of pain after childbirth | Taken while cooked and mixed with boga leaves  Decoction of leaves taken orally | Backache, Hydrocele, Joint Pain, Rectum ulcers, and Conjunctivitis | (Chhabra et al., 1987; Ramathal and Ngassapa, 2001) |
|  | *Adhatoda engleriana* (Lindau) C.B. Cl | Tugutu (Sambaa) | 185, 284 | Tanga | Leaves | To remove pain in childbirth | Leaves decoction taken orally | Laxative, Emetic, Swollen liver, spleen, Nausea and Tuberculosis | (Hedberg and Hedberg, 1982) |
|  | *Justicia salvioides* Milne Redh. | Muluguti | Augustino 151 | Tabora | Roots | Infertility | Oral |  | (Augustino et al., 2011) |
| Agavaceae | *Dracaena deremensis* Engl. | Isale | 3087 |  | Root | Quicken birth once labour has began | Roasted roots taken orally | Hepatomegaly, Rheumatism and Malaria | (Chhabra et al., 1984) |
| Aizoaceae | *Zaleya pentandra* (L) Jeffrey | Isindura (Jita & Ruri) Kaitongo (Sukuma) | SMM-BD25 | Mara | Whole plant | To shorten labour pains during delivery Abortifacient Dysmenorrhoea Polymenorrhoea | Powdered whole plant, Decoction of roots taken orally | Athlete's foot, Septic wounds, Wounds and Stomach pain | (Maregesi et al., 2007a) |
| Asphodelaceae | *Aloe sp.* | Aloe vera (Swahili), Enkaka |  | DSM, Kagera | Leaves | induce abortion, Menstruation | Decoction of leaves mixed with *Azadirachta indica* and *Vernonia amygdalina* taken orally |  | (Lindh, 2015; Nikolajsen et al., 2011; Rasch et al., 2014) |
|  | *Aloe lateritia* | Lichingiri/ Litembo |  | Morogoro, Iringa | Roots | Delayed birth | A decoction of roots is taken orally |  | (Shangali et al., 2008) |
|  | *Aloe vera* L. |  |  | Morogoro |  | Used by breastfeeding mother as Galactagogue |  |  | (Millinga et al., 2022) |
| Amaryllidaceae | *Crinum papillosum* Nordal | Mfunguo, Ekiwakye kitunguru | 1828, MJM 3236 | Morogoro, Kagera | Root, Tubers | Against prolapse of the uterus, for treating swollen breast | Decoction of root taken orally. The underground tuber is mixed with anthil soil | Cancer, Swollen legs and abdomen | (Chhabra et al., 1987; Moshi et al., 2012) |
|  | *Scadoxus multiflorus* (Martyn) Raf. | Ihemba-nyoka (Pale) Mbegere (Ndengereko) | 3115 | Pwani | Root | Against sterility in females | Decoction of root taken orally | Menal illiness | (Chhabra et al., 1987) |
|  | *Allium cepa* |  |  | Mwanza |  | labor induction |  |  | (Dika et al., 2017) |
|  | *Allium sativum* L |  |  | Morogoro |  | Used by breastfeeding mother as Galactagogue |  |  | (Millinga et al., 2022) |
| Anacardiaceae | *Lannea stuhlmanii* Engl. | Mumbu (Zigua), Sayu (Sukuma), Mtundutwa (Pare) | 1822, 2448 2992 | Morogoro Pwani Kilimanjaro | Roots, Leaves | Against sterility in females Hasten child birth | Decoction of root taken orally Decoction of leaves | Stomachache, Fever, Dysentry, Asthma, culosis, Anemia, Antibacterial | (Chhabra et al., 1987) |
|  | *Ozoroa mucronata* (Krauss) | Muungwae (Ndengereko) Ngombo kilangu (Zaramo) Mkalakala (Zigua), Mkamachumu (Pwani) | 580, 1966 2389 | DSM, Pwani | Rootbark, roots | Against sterility in females, Induces abortion, Infertility | Powdered rootbark mixed with kimelelandembo, mixed with honey diluted with water, Dicoction; Oral | Fever, Cough, Gonorrhoea, Impotency, Chronic ulcers, kidney plumes, Intestinal parasites, Bilharzia, Snakebite | (Abdallah et al., 2007; Chhabra et al., 1987) |
|  | *Rhus longipes* Engl. | Mpungu (Pare) | 3089 | Kilimanjaro | Root | Induces abortion | Decoction of root taken orally | cancer, Indigestion, Malaria, Infuenza | (Chhabra et al., 1987) |
|  | *Sorindeia madagascariensis* DC | Mpilipili (Swahili) Mkunguma (Sambaa) | 964, 1960, 184 | Pwani, Tanga | Root | Against vagina prolapse Against menorrhalgia Against polymenorrhalgia Against menstrual problems | Decoction of root taken orally | Pneumonia, Cold, Fever, Chest pain, Abdominal pain, Venereal sores, Aphrodisiac, Constipation, Gonorrhoea, Diarrhoea, Edema and ascites, Mental illness, Malaria, Hookworm, Hemorrhoids, Syphilitic sores | (Chhabra et al., 1987; Hedberg and Hedberg, 1982) |
|  | *Rhus vulgaris* Meikle | Umuragara (Kinyarwanda) |  | Kagera | Leaves | Oxytocic action Against infertility To ease delivery | Fresh leaves are burned and the ash is used | Scabies, Diarrhoea, Wounds, Gonorrhoea | (Ramathal and Ngassapa, 2001) |
|  | *Lannea schweinfurthii* var. stuhlmannii (Engl.) Kokwaro Engl. | Mtundutwa, Mugumbu | 2992 | Pwani | Leaves, Roots | Relieve abdominal pains, Hasten child birth, Menorrhagia | Infusion of leaves taken orally, decoction of root | Anemia, Headache, Abdominal pains, Polio, Carbuncles and Absesses | (Abdallah et al., 2007; Chhabra et al., 1984) |
|  | *Rhus natalensis* Bernh. ex Kraus | Muhungulu (Zigua), Mkumbambogo (Digo), Mfunguzi | 2145, 28, RMK045 | Pwani, Tanga, Kigoma | Root, Leaves | Against polymenorrhoea Against dysmenorrhoea, Aborticide, Against repeated still-births, Enhance fertility | Decoction of root taken orally, Roots eaten with chicken soup, Dried powdered leaf cold infusion is taken orally. | Pain in subumblical area Headache and neck pain, Constipation, Laxative Antimalarial, Influenza Abdominal pains, Gonorrhoea, Hookworm Fits in children . | (Chhabra et al., 1987; Hedberg and Hedberg, 1982; Kingo and Maregesi, 2020) |
|  | *Ozoroa insignis* Del. Susbsp. Reticulata (Bak.f) Gillet | Muhombe(Zigua), Mwembe pori | 208, Augustino 198, ITM 3709 | Tanga, Tabora | Roots, ;Leaves, Barks | Galactogogue, Antiabortion | Roots decoction taken orally | Bilharzia, Malaria, Aphrodisiac, Diarrhoea, Stomach pain, Dysentry, Pink eye, haemorrhoids, anal eczema, epilepsy, gonorrhoea, | (Augustino et al., 2011; Hedberg and Hedberg, 1982) |
|  | *Searsia longipes* (Engl.) Moffett | Olmesigei |  | Pwani | Root | Menorrhagia | Decoction; oral |  | (Abdallah et al., 2007) |
|  | *Mangifera indica* L | Mwembe |  | Morogoro, Iringa | Roots | Infertility |  | Tuberculosis, Dysentery, Waist pain | (Augustino and Gillah, 2005) |
| Annonaceae | *Uvaria acuminata* Oliv | Msofu (Swahili), Mungwene, Mngwene, Msalansi (Digo) | 1150, 9 18 89 | Pwani, Tanga | Root | Against menorrhagia, Against painful menstruation | Decoction of root taken orally | Abdominal pain, Cough Dysentery, Pectoral disease, Mental illness, Convulsions, Dysentry and Snake bite | (Chhabra et al., 1987; Hedberg and Hedberg, 1982) |
|  | *Annona senegalensis* Pers. subsp. senegalensis. | Mbokwe, Mtopetope (Digo), Mukonola, Mutopetope | 22, 48, 66, 71, 78, Augustino 144 | Tanga, DSM, Tabora, Pwani | Roots | Against sterility in women, To wean a child from its mother's breast, Pregnancy, Labour progression, Polymenorrhoea | Roots decoction taken orally | Abdominal pain, Snake bites, Abscesses, Wounds, Gonorrhoea Expectorant, Tumours Colds, Sleeping sickness, Diarrhoea, Respiratory diseases, Eye diseases Ear disease ad Ulcers | (Abdallah et al., 2007; Augustino et al., 2011; Hedberg and Hedberg, 1982; Lindh, 2015) |
|  | *Voacanga africana* Stapf. | Mberebere(Sambaa, Pare) | 283 | Kilimanjaro | Roots | Against frequent menstruation Against dysmenorrhoea Hypotensive | Root powder mixed with porridge | Internal sores Frequent urination in men Spasms of the heart | (Hedberg and Hedberg, 1982) |
|  | *Xylopia odoratissima* Oliv. | Mushenene | Augustino 175, ITM 3707 | Tabora | Roots, leaves | Infertility | Oral, nasal | Stomach ache, diabetes, abdominal ulcers, fever, epilepsy | (Augustino et al., 2011) |
|  | *Cocos nucifera* L. | Mnazi |  | Pwani | Roots | Amenorrhoea | Decoction; oral |  | (Abdallah et al., 2007) |
|  | *Friesodielsia obovata* (Benth.) Verdc. Syn of *Monanthotaxis obovata* (Benth.) P.H. Hoekstra | Msalasi, Musalasi, Msasi | Augustino 170 | Morogoro, Tabora | Roots | Infertility, placenta expulsion | Oral | stomach ache, anaemia, snakebite | (Augustino et al., 2011; Augustino and Gillah, 2005) |
| Apiaceae | *Steganotaenia araliacea* Hochst. | Mnyongamembe |  | Mbeya | bark, root | To egulate menstrual cycle |  |  | (Hilonga et al., 2019) |
| Apocynaceae | *Ancylobothrys petersiana* Pierre | Kilela (Zaramo) Luneke (Nguu) | 360 569 | Pwani | Root | Against painful menstruation | Roots cooked with chicken (soup) | Constipation | (Chhabra et al., 1987) |
|  | *Diplorhynchus condylocarpon* (Muell.Arg.) Pichon | Mtogo (Bena,Swahili,Zigua) Mulondo(Zaramo) | 70 3048 | Pwani | Root | Against painful menstruation Against frequent abortion Against sterility in females | Root infusion taken orally Decoction of root taken orally | Gonorrhea, Fever, Pneumonia, Leprosy Abdominal pain and Cough | (Chhabra et al., 1987) |
|  | *Rauvolfia caffra* Sond | Msesewe (Chagga) | 2980 | Kilimanjaro | Root | Against irregular menses | Decoction of root taken orally | Blood pressure, Abdominal pain, Constipation, General swelling, eumatism and Pneumonia | (Chhabra et al., 1987) |
|  | *Gymnema inodorum* (Lour.) Decne. | eghekómóóri/ ibhikómóóri |  | Mara | Leaves | Dysmenorrhea (menstrual cramps) | Boil in water, taken orally |  | (Charwi et al., 2023) |
|  | *Rauvolfia mannii* | Nyavihongo |  | Morogoro, Iringa | Roots | Infertility | A decoction from the roots is taken orally for seven days |  | (Shangali et al., 2008) |
|  | *Catharanthus roseus* (L.) G.Don | Stars of Jerusalem |  | Morogoro | Flowers | Pregnacy High Blood Pressure |  |  | (Augustino and Gillah, 2005) |
| Aquifoliaceae | *Ilex mitis* | Mhangavya/ Kihaga |  | Morogoro, Iringa | Roots | Infertility | A decoction from the roots is taken orally for seven days |  | (Shangali et al., 2008) |
| Araliaceae | *Cussonia zimmermannii* Harms | Mtindi (Sambaa) | 110 | Pwani | Root, Stem bark | Against postpartum hemorrhage Induces labour | Roots together with roots of munyonyo cooked with chicken and the soup is drunk | Hypertensive-encephalopathy, Mental illiness, Gonorrhoea, Malaria, and Epilepsy | (Chhabra et al., 1987) |
|  | *Cussonia arborea* Hochst. ex A. Rich. | Mgagigagi |  | Pwani | Roots | Infertility, Menorrhagia | Decoction; oral |  | (Abdallah et al., 2007) |
| Asclepiadaceae | *Parquetina nigrescens* (Afz.) Bullock. | Sikombe (Makonde). | 149 | Tanga | Whole plant | Against dysmennorhoea |  | Aphrodisiac | (Hedberg and Hedberg, 1982) |
| Asparagaceae | *Dracaena steudneri* Engl | Mgologolo | MJM 3169 | Kagera | Leaves | Against infertility in women | The leaves are burnt and the ash is then combined with soda ash and the powder licked | Hernia, Splenomegaly Asthma and Chest problems in children | (Moshi et al., 2012) |
|  | *Asparagus falcatus* L | Olopirolopapaa |  | Morogoro | Root | Infertility |  |  | (Hilonga et al., 2019) |
| Asteraceae | *Vernonia lasiopus* O Haffm | Itughutu (Pare) Mtugutu (Chagga), Mhasha (Sambaa) | 2993 174 281 | Kilimanjaro, Tanga | Leaves, roots | Against stomach-ache during periods, Against sterility in females, To purify the milk of lactating women or to produce lactation, To facilitate childbirth, and Given when a woman cannot be pregnant in association with menstrual bleeding. | Decoction of leaves taken orally, Decoction of root taken orally Chewed leaves | Stomach-ache, Sex stimulant in males, Stomach pains, Aphrodisiac, Purgative and Epilepsy | (Chhabra et al., 1989, 1984; Hedberg and Hedberg, 1982) |
|  | *Conyza stricta* Willd | Mterezi (Vidunda) | 3182 | Morogoro | Roots | Against female sterility | Decoction of root is mixed with porridge and taken orally |  | (Chhabra et al., 1989) |
|  | *Crassocephalum bojeri* (DC.) Robyn | Ihorohoro (Chagga) | 3010 | Kilimanjaro | Leaves | Against nausea in pregnant women | Decoction of leaves taken orally |  | (Chhabra et al., 1989) |
|  | *Microglossa pyrifolia* (Lam.) O. Kuntze | Mfurufuru (Zaramo) Mlenga (Zigua) | 283 570 | Pwani | Roots | Against uterine prolapse | Decoction of root taken orally | Hernia | (Chhabra et al., 1989) |
|  | *Pluchea dioscoridis* L DC | Mnyenywa (Zigua) Mnywenywe (Swahili) | 554 1078 1433 3126 267 | Pwani, Tanga | Leaves, rootbark | Against dysmenorrhoea, Against sterility in women | Leaf decoction mixed with tea or porridge, Decoction of root bark and cooking fat (a few drops) taken orally | Hernia, Fever, Backaches Skin disease, Sterility in men and Stimulant Infantile ailements | (Chhabra et al., 1989; Hedberg and Hedberg, 1982) |
|  | *Crassocephalum vitellinum (*Benth.) S. Moore. | Uwenge (Sambaa), Umusununu (Kinyarwanda) | 172 | Tanga | Roots, Leaves | To improve the quality of the milk in a lactating woman, Used for abortion | Decoction of roots and leaves in combination with roots of Mdagha taken orally, Infusion of the leaves taken orally | Body & mouth sores Eye disease, Gonorrhoea, Constipation and Sores | (Hedberg and Hedberg, 1982; Ramathal and Ngassapa, 2001) |
|  | *Vernonia jugalis* Oliv. & Hiem. | Mhasha (Sambaa) | 176 | Tanga | Roots | To promote birth | Decoction of root taken orally | Stomach troubles Epilepsy | (Hedberg and Hedberg, 1982) |
|  | *Vernonia usambarensis*. Hoffm | Mpalwe (Pale) | 282 | Kilimanjaro | Roots | Against excessive menses | Decoction of root taken orally |  | (Hedberg and Hedberg, 1982) |
|  | *Senecio discifolius* Oliv. | Imangwe (Ruri) | SMM-BD09 | Mara | Whole plant | Against stomachache during pregnancy Stimulation of milk after child birth |  | Syphilis Sore eyes | (Maregesi et al., 2007a) |
|  | *Ageratum conyzoides* (L.) L | Omwigara | MJM 3211 | Kagera | Leaves, Roots | Against fibroids in women For those with difficulties to conceive | Rooots are chewed fresh Decoction of leaves taken orally | Cough remedy, Constipation, Peptic ulcers and Antispasmodic | (Moshi et al., 2009) |
|  | *Vernonia amygdalina* Delile | Omubirizi, Omubabazi no.2, Kilulungunja | VR nr. 10, RMK018 | Kagera, Kigoma | Leaves, Stalk | Induction of abortion | Infusion of plant taken orally; Fresh leaves decoction is taken orally. | Typhoid, Malaria | (Kingo and Maregesi, 2020; Nikolajsen et al., 2011; Rasch et al., 2014) |
|  | *Bidens pilosa* L | Akakurura | VR nr 12 | Kagera | Roots | Induction of abortion | Roots are chewed |  | (Nikolajsen et al., 2011; Rasch et al., 2014) |
|  | *Solanecio mannii* (Hook.f.) C. Jeffrey | Umutagara (Kinyarwanda) |  | Kagera | Leaves | Oxytocic action | Infusion of the leaves taken orally |  | (Ramathal and Ngassapa, 2001) |
|  | *Guizotia scabra* (Vis.) Chiov | Igishikashike (Kinyarwanda) |  | Kagera | Leaves | Used for abortion | Ash of burnt leaves is taken orally | Liver disease Intestinal worms Diarrhoea | (Ramathal and Ngassapa, 2001) |
|  | *Helichrysum odoratissimum* Less. | Isinunu (Kinyarwanda) |  | Kagera | Leaves | Against postpartum bleeding | Fresh leaves are ground, placed in hot water and the infusion is taken orally | Coughs Colds | (Ramathal and Ngassapa, 2001) |
|  | *Berkheya bipinnatifida* (Harv.) Roessler |  |  | Pwani |  | Menorrhagia |  |  | (Abdallah et al., 2007) |
|  | *Vernonia subuligera* O.Hoffm. & Engl. Syn: *Gymnanthemum myrianthum* (Hook.f.) H.Rob. | Mtugutu |  | Morogoro | Roots | Women stomach ache |  |  | (Augustino and Gillah, 2005) |
|  | *Sphaeranthus sp* | Mtibu | RMK027 | Kigoma | Leaves | Dysmenorrhoea. | Fresh leaf decoction is taken orally. |  | (Kingo and Maregesi, 2020) |
| Balanitaceae | *Balanites aegyptiaca* (L.) Del | Liluguyu (Jita) | SMM-BD27 | Mara | Whole plant | Abortifacient |  | Asthma Dry cough Chest infection Antipaasitic Antipyretic Fish poison | (Maregesi et al., 2007a) |
| Berberidaceae | *Berberis holstii* Engl. | Kimakatsa |  | Morogoro, Iringa | Roots | Infertility | A decoction from the roots is taken orally for seven days |  | (Shangali et al., 2008) |
| Bignoniaceae | *Kigelia africana* (Lam.) Benth | Lisamwa (Jita) Ng’wicha (Sukuma), Myegea | SMM-BD03, 52 141 | Mara, Morogoro | Root, Stembark, Baks | Against aneamia especially with pregnant women, Against excessive mestrual bleeding, Applied externally on a woman’s breasts to produce lactation, Infertility. | Fruit is boiled and taken orally, Roots decoction taken orally | Pneumonia, Gonorrhea, Bilharzia, Dysentery, Coughing, Female gynecological problems Malaria, Syphilis, Male sterility, Aphrodisiac and Enlargment of sexual organs | (Augustino and Gillah, 2005; Hedberg and Hedberg, 1982; Maregesi et al., 2007a) |
|  | *Ehretia amoena* Klotzsch | Nembu, Jabalelon, Onjanjokuo |  | Morogoro, Mbeya | Root | Dysmenorrhoea |  | Eyeproblems,stomachache ,gonorrhea,bilhaziasis | (Hilonga et al., 2019) |
|  | *Markhamia obtusifolia* (Bak.) Sprague | Mubapa | Augustino 123 | Tabora | Roots | Infertility | Oral, bath, massage | Aphrodisiaclove, lucky | (Augustino et al., 2011) |
| Boraginaceae | *Ehretia amoena* Klotzsch | Mkirika, Msimbampuku(Zigua) Lipepele (Ngoni) Mchirika (Dunda) Msasananda (Kaguru) Nkatakata (Makonde) | 1767, 203 | Pwani, Tanga | Root, Leaves, Steambark, Barks | Against poly menorrhagia, Polymenorrhea, Againt lower abdominal pain Women to conceive, Induce menstruation, Against painful menstruation, Against miscariage | Decoction of root taken orally, Stembark decoction taken orally | Herina, Rectal prolapse Epilepsy, Internal swellings, Bleeding (nose,mouth,ear)Covulsion Pneumonia, Tuberculosis, Gonorrhoea, Bilharzia, Hook worm, Lower abdominal pain., Vomitting, Wounds and Mental illness | (Abdallah et al., 2007; Chhabra et al., 1987; Hedberg and Hedberg, 1982) |
|  | *Heliotropium subulatum* (DC.) Martelli | Msasananda (Kaguru) | 2909 | Morogoro | Leaves | Induces menstruation Against post-parturation disease | Infusion of leaves taken orally |  | (Chhabra et al., 1987) |
| Burseraceae | *Commiphora africana* (A. Rich.) Eng | Malamula, Mntwintwi (Zigua), Mpome (Ngindo), Mrimbwi (Makua), Muntonto, Esilalei | 33 1218 | Pwani, Tabora | Stem bark, Roots, bark | Against dysmenorrhoea, Againstmenorrhagia, Preventabortion, Prevent cervical prolapse, Infertility | Decoction of rootbark taken orally | Dysentery, Blood diarrhoea, Gonorrhoea, Bilharzia, Leprosy, Athritis , Vomitting, Aphrodisiac, Diabetes, trachoma, snakebite | (Augustino et al., 2011; Chhabra et al., 1987) |
|  | *Commiphora pteleifolia* Engl. | Mtuntwi (Zigua) | 561 | Pwani | Roots | Against cervical prolapse | Decoction of rootbark taken orally | Rectal prolapse | (Chhabra et al., 1987) |
|  | *Commiphora madagascariensis* Jacq. | Mtonto (Zigua) | 215 | Tanga | Roots | Against excessive menstruation | Decoction of roots taken orally | Abdominal pain, Fever and Toothache | (Hedberg and Hedberg, 1982) |
|  | *Commiphora boiviniana* Engl. | Danda chindi (Digo) | 69 | Tanga | Stembark | Warm stem bark is rubbed on the breasts to produce lactation | Warmed stem bark | Gonorrhoea, Hydrocele, Aphrodisiac, Dysentry and Indigestion | (Hedberg and Hedberg, 1982) |
| Canellaceae | *Warburgia sp.* | Mwifu (Zigua/Sambaa) |  | DSM |  | Abortion |  |  | (Lindh, 2015) |
|  | *Commiphora mollis* (Oliv.) Engl. | Mponda |  | Morogoro | root | infertility |  | Stomachache | (Hilonga et al., 2019) |
|  | *Warburgia stuhlmannii* Engl. | Sokonoi |  | Dodoma and Morogoro | bark, root | dysmenorrhoea |  | Headache, hernia, malaria,diabetes, Libido disoder,numbness,cold and flu ,fever | (Hilonga et al., 2019) |
| Cannabaceae | *Trema orientalis* (L.) Blume | Mbefu/ Mhafu |  | Morogoro, Iringa | Roots | Delayed birth | A decoction of roots is taken orally |  | (Shangali et al., 2008) |
| Cannaceae | *Canna indica* L. | Amarango/ Embakyo | MJM 3172 | Kagera | Leaves | Treatment of infertility in men and women Makes a woman conceive easily Regulation of fertility | The leaves are dried, groud and the powder is then used or pounded, soaked in a small amount of water and a patient given a spoonful |  | (Moshi et al., 2012) |
| Capparidaceae | *Capparis tomentosa* Lam | Mtungulang'osa (Zigua) | 2780 | Pwani | Root | Against barrenness | Decoction of root taken orally | Gonorrhoea, Bilharzia Cough, Pneumonia, Impotency, Asthma and Snake bites | (Chhabra et al., 1989) |
|  | *Maerua kirkii* (Oliv) F. White | Msaka (Zigua) | 207 | Pwani | Root | Induces labour | Decoction of root taken orally | Asthma, Limb pain, Stomach disorders and Antibacterial | (Chhabra et al., 1989) |
|  | *Gynandropsis gynandra* (L.) Briq | Isogi (Kinyarwanda) |  | Kagera | Leaves | Used to ease childbirth | Decoction of leaves taken orally | Chest infections | (Ramathal and Ngassapa, 2001) |
| Caricaceae | *Carica papaya* L | Mpapai dume |  | Morogoro | Fruits/ Roots | Women stomach ache |  |  | (Augustino and Gillah, 2005) |
| Celastraceae | *Elaedendron schweinfurthianum* Loes. | Mnenekanda (Zigua) | 440 | Pwani | Rootbark, Stem bark | Against menstrual disorders, Against menorrhagia | Decoction of root bark taken orally, Infusion of stem bark taken orally | Syphillis, Coughing blood Constipation, Diarrhoea and Antimicrobial | (Chhabra et al., 1989, 1984) |
|  | *Elaeodendron buchananii* (Loes) Loes. | Mnenekanda (Zaramo) | 187 | Pwani | Root | Against menorrhagia Against female infertility | Decoction of root taken orally | Constipation, Diarrhoea, Abdominal pain, Male impotency, Wounds, Syphilis and Coughing blood | (Chhabra et al., 1989) |
|  | *Maytenus mossambicensis* (Klotzsch) Blakelock | Mwambangoma (Zigua) | 312 | Pwani | Root | Against female sterility Regulate menstrual cycle | Root decoction mixed with porridge | Malaria | (Chhabra et al., 1989) |
|  | *Maytenus senegalensis* (Lam) Exel | Mwambangoma (Zigua), Mwezya, Lweja | 11, Augustino 101 | Pwani, Tabora | Leaves, Roots, bark | Against sterility in females; Infertility | Leaves decoction taken orally | Pneumonia, Rheumatism, Fever, Snake bite, Severe abscesses, Bilharzia, Gingivitis, Dental carries, and Diarrhoea | (Augustino et al., 2011; Chhabra et al., 1989) |
|  | *Salacia bussei* Loes | Mbwiki (Zigua) Mshakii (Ndengereko) | 2 | Pwani | Roots, Seeds | Against menorrhagia | Decoction of root and three fresh seeds of Zea mays taken orally | Fever | (Chhabra et al., 1989) |
|  | *Salacia madagascariensis* (Lam.) DC. | Mbwiki (Zigua) Mshakii (Ndengereko) | 1399 | Pwani | Roots, Seeds | Against menorrhagia | Decoction of root and three fresh seeds of Zea mays taken orally | Fever | (Chhabra et al., 1989) |
|  | *Maytenus putterlickioides* (Loes.) Exe11 & Mendonca. | Mtulavuha (Zigua) | 211 234 | Tanga | Roots | Againt irregular menstruation Against dysmenorrhoea | Decoction of roots mixed with Mmorwe taken orally | Hernia, Swollen testicles, Aphrodisiac, Internal body injuries and Antiemetic | (Hedberg and Hedberg, 1982) |
|  | *Elaeodendron schlechterianum* (Loes.) Loes | Chihusilo (Jita) Ngakama (Sukuma) | SMM-BD16 | Mara | Rootbark | Against dysmenorrhoea, Against female infertility | Root bark decoction taken orally | Wounds, Anaemia, Hypertension, Male impotence, Abcesses and Carbuncles | (Maregesi et al., 2007b) |
|  | *Maytenus heterophylla* (E&l. & Zeyh.) N. Robs. | Ndegamau (Makonde) Mjengamanyigu (Swahili) | 147 | Tanga | Leaves | Against dysmenorrhoea | Decoction of leaves taken orally | Epilepsy, Abcesses, Anthelmintic, Hernia and Syphilis | (Hedberg and Hedberg, 1982) |
| Chenopodiaceae | *Chenopodium ambrosioides* L. | Mdiwasoko (Chagga), Injaga-yabekwabi (Jita) Nemu ya Masai (Sukuma) | 2972, SMM-BD36 | Moshi, Mara | Whole plant, Leaves | Abortifacient, Against menstrual disorders Against dysmenorrhea |  | Anthelmentic, Asthma, Intestinal ulcers, Eczema, Erysipelas Diaphoretic, Oral thrush Vaginal ulcers, Cancers, Antidiabetic and Antiamoeba | (Chhabra et al., 1989; Maregesi et al., 2007b) |
|  | *Chenopodium opulifolium* Schrad. Ex Koch & Ziz | Ufunguo (Vidunda), Mogabhogole (Jita) | 2905, SMM-BD33 | Morogoro, Mara | Roots | Induce menstruation Hasten birth | Decoction of root taken orally | Antibacterial, Fungal/bacterial scalp infection, Female asthenia and Abdominal colic for newborns | (Chhabra et al., 1989; Maregesi et al., 2007b) |
|  | *Atriplex sp* | Mfunguo (Swahili) |  | DSM |  | Right before birth |  |  | (Lindh, 2015) |
| Chrysobalanaceae | *Parinari curatellifolia* Benth. | Mumbula | Augustino 156 | Tabora | Roots | Infertility | Oral |  | (Augustino et al., 2011) |
| Cleomaceae | *Cleome gynandra* L. | urusáágha ichinsáágha |  | Mara | Leaves | Pain during the menstruation period | Boil in water, Oral | Pneumonia Ear problems, it adds nutrients to fight against diabetes, heart attacks, cancer, and headache | (Charwi et al., 2023) |
| Combretaceae | *Combretum molle* G. Don | Mlama (Swahili) Mnana (Zigua) | 317 521 121 244 | Pwani, Morogoro | Roots | Against sterility, Abortifacient, Aid childbirth, Women's stomach ache, Infertility | Decoction of root taken orally | Hematuria, Diarrhoea, Abdominal pain, Expectorant, Hookworms, Leprosy Snake bite, Fever, Dysentry, General body swellings, Abdomen swelling, Constipation and Antitumour activity | (Augustino and Gillah, 2005; Chhabra et al., 1989; Hedberg and Hedberg, 1982) |
|  | *Pteleopsis myrtifolia* (Laws.) Engl. & Diels | Kakongwa, Mneke, Mpelepele (Ngindo), Mgovu (Zaramo), Mgonji (Zigua) | 342 | Morogoro, Tanga | Roots | Against menorrhagia, Against infertility Against abortion | Decoction of root taken orally | Venereal diseases Dysentry | (Chhabra et al., 1989; Hedberg and Hedberg, 1982) |
|  | *Combretum collinum* Fresen. | Mnama (Pare), Mlama mweupe | 135 268 | Tanga, Morogoro | Roots | Against excessive menstrual bleeding, Women's stomach aches | Decoction of roots taken orally | Diarrhoea, Abdominal pain, Gonorrhoea, Rectal prolapse Malaria, Dysentry and Snake bite | (Augustino and Gillah, 2005; Hedberg and Hedberg, 1982) |
|  | *Combretum zeyheri* Sond. | Mlamamweupe (Zigua), Musana | 210, Augustino 172, ITM 3706 | Tanga, Tabora | Stembark, Roots, leaves, Bark | Arrest menstrual flow, Infertility | Powdered stembark in vagina; Oral | Diarrhoea, Vomiting, Dysentry, Toothache, Cough and Scorpion bite | (Augustino et al., 2011; Hedberg and Hedberg, 1982) |
|  | *Combretum obovatum* F. Hoffm. | Vugoweko | Augustino 210 | Tabora | Root | Infertility, antiabortion | Oral, massage, bath | Abscess, love, gonorrhea, snakebite | (Augustino et al., 2011) |
|  | *Combretum apiculatum* Sond. | Mlamadori |  | Pwani | Root | Menorrhagia | Decoction; oral |  | (Abdallah et al., 2007) |
|  | *Terminalia sambesiaca* Engl. & Diels | Mpululu |  | Morogoro | Roots | Infertility |  |  | (Augustino and Gillah, 2005) |
| Commelinaceae | *Commelina benghalensis* L. | Makengera (Chagga) Makengeria (Pare) Mzimakalio (Swahili) | 2983 | Moshi | Roots | Against female sterility | Decoction of root is warmed with camphor and taken orally | Gonorrhoea, Conjuctivitis, Burns, Delmucent, Laxative, Emollient, Leprosy and Sore throat | (Chhabra et al., 1989) |
|  | *Commelina africana* L | Eitezi | VR nr.2 | Kagera | Leaves, Stems | Induction of abortion | Pounded plant is inserted in the vagina |  | (Nikolajsen et al., 2011; Rasch et al., 2014) |
| Connaraceae | *Agelaea pentagyna* (Lam.) Baill. | Nyakabiki/ Mlungamo |  | Morogoro, Iringa | Roots | Infertility | A decoction from the roots is taken orally for seven days |  | (Shangali et al., 2008) |
| Cucurbitaceae | *Cucurbita moschata* Duchesne. | Maboga | EA042 | Morogoro | Root | Expulsion of placenta; | infusion taken orally |  | (Amri and Kisangau, 2012) |
|  | *Zehneria scabra* (Linn. F.) Sond. | Engenyi | PHS nr. 51 | Kagera | Leaves, Stems | Induction of abortion | Leaves and stems decoction taken orally |  | (Nikolajsen et al., 2011; Rasch et al., 2014) |
|  | *Momordica foetida* Schum. & Sond | Umwinuiwa/ Umwishwa (Kinyarwanda), Ruhunduhundu (Zaramo) |  | Kagera, Pwani | Leaves | Abortifacient, Induce labour | Infusion of the leaves taken orally | Constipation, Roundworms, Purgative, Emetic, Purgative, Gout, Fever, Hemorrhage, Epilepsy, Intestinal, Earache, Insect bite, Snake bite, Malaria and High blood pressure | (Chhabra et al., 1989; Ramathal and Ngassapa, 2001) |
|  | *Cucurbita pepo* L |  |  | Morogoro | Seeds | Used by breastfeeding mother as Galactagogue |  |  | (Millinga et al., 2022) |
|  | *Tefairia pedata* |  |  | Tanga |  | Improve lactation after childbirth | Decoction; oral |  | (Kideghesho and Msuya, 2010) |
|  | *Momordica charantia* L. | Zukini |  | Pwani | Leaves | Menorrhagia | Decoction; oral |  | (Abdallah et al., 2007) |
| Dichapetalaceae | *Dichapetalum stuhlmannii* Engl | Mdimu-pori (Swahili) | 1696 | Pwani | Bark | Introduce labour | Powdered bark is mixed with porridge | Blood diarrahoea Headache | (Chhabra et al., 1989) |
| Dilleniaceae | *Tetracera boiviniana* Baill | Kilio, Mpinga (Ndengereko) Mpalafigi (Swahili) Mpitayo (Zaramo) | 263 1242 2312 | Pwani | Roots | Against menorrhagia Inducing labour pains | Decoction of root taken orally | Stomach pains, anasarca, Gonorrhoea Hernia Snake bite, Diarrhoea and Leprosy | (Chhabra et al., 1989) |
| Ebenaceae | *Diospyros usambarensis* F. White | Kinyalinyali, Mgoto, Mwiza (Zigua) Mnake (Ngindo) Mwiloiloi (Kimbo) | 64 159 1693 | Morogoro Pwani DSM | Roots | Cervical prolapse | Decoction of root taken orally | Constipation, Ereptions Rashes, Epilepsy, Malaria, Psychriatic Problems, Joint pains, Stomach pain, Measles, Snake bites and Wounds | (Chhabra et al., 1989) |
|  | *Euclea natalensis* A. DC | Mdala (Zigua) Mlamamwitu (Swahili) Mnindimya (Matumbi) | 309 1575 2256 | Pwani | Roots | Against dysmenorrhoea Against polymenorrhoea | Decoction of root taken orally | Constipation, Dysuria Abdominal pain, Diarrhoea, Skin diseases, Heart pain, and Ancylostomiasis | (Chhabra et al., 1989) |
|  | *Euclea racemosa* Murr. Subsp. Schimperi (A.DC.) F. White | Mdala (Zigua) Tuku (Pare) | 99 3111 | Pwani, Kilimanjaro | Roots | Against spasmodic dysmenorrhoea | Powdered root mixed with porridge | Cancer, Constipation, Abdominal pains, Splenic pains, Purgative, Ancylostomiasis and Abdominal pains | (Chhabra et al., 1989) |
|  | *Diospyros fischeri* Gürke | Mfubata | Augustino 117 | Tabora | Roots, leaves | Placental removal after delivery | Oral | Stomach ache, earache, snakebite, wounds | (Augustino et al., 2011) |
|  | *Euclea divinorum* Hiern. | Mdaa | Augustino 116 | Tabora | Roots, leaves | Infertility | Oral | wounds, snakebite | (Augustino et al., 2011) |
| Euphorbiaceae | *Antidesma venosum* Tul | Inyamaza | 2904 |  | Roots | Against prelapse of uterus | decoction taken orally | Emetic Snake bite Liver complaints Abdominal pain Hook worms | (Chhabra et al., 1984) |
|  | *Synadenium glaucescens* Pax | Mwasa | 3097 |  | Leaves | Against excesive menses | Infusion of leaves taken orally |  | (Chhabra et al., 1984) |
|  | *Ricinus communis* L. | Nyonyo (Swahili), Omujuna, Omudimu, Mbarika, Mkale | VR nr. 5, Augustino 114 | DSM, Kagera, Tabora | Roots, Leaves | Anti-conception, Menstruation, Induction of abortion, Labour pains, placenta expulsion | Decoction of roots taken orally | Stroke | (Augustino et al., 2011; Lindh, 2015; Nikolajsen et al., 2011; Rasch et al., 2014) |
|  | *Euphorbia tirucalli* L. | Minyaa (Swahili) Masongorwa (Jita, Sukuma) | SMM-BD43 | Mara | Young branches | Against women sterility |  | Ophthalmic infections, Gonorrhea, Syphilis, Sore throat, Stomach complaints, Malariaand Snake bite | (Maregesi et al., 2007b) |
|  | *Bridelia micrantha* (Hochst.) Baill | Mshamako | MJM 3166 | Kagera | Roots | Against amenorrea Against dysmenorrhea | Decoction of roots taken orally | Antimalarial Antibacterial | (Moshi et al., 2012) |
|  | *Euphorbia hirta* L | Kahyebulimbe, empango, Lonzwe, Vakikulu | MJM 3184, Augustino 112 | Kagera, Tabora | Aerial parts, Roots, leaves, Bark | Galactagogue, menstrual disorders | Decoction of aerial parts taken orally | Hernia, hypertension, convulsion, epilepsy, Warts, Cataracts, Diuresis | (Augustino et al., 2011; Moshi et al., 2012) |
|  | *Euphorbia mossambicensis* (Klotzsch & Garcke) Boiss. | Kahyebulimbe Enkye | MJM 3185 | Kagera | Leaves | Galactagogue | Decoction of leaves taken orally | Cataracts | (Moshi et al., 2012) |
|  | *Jatropha curcas* L. | Ekiho | MJM 3187 | Kagera | Leaves | Against mastitis | The leaves are baked under fire and the sap squeezed into the affected area for 5 days. | Koilonichia Antibacterial | (Moshi et al., 2012) |
|  | *Manihot esculenta* Crantz | Cassava, Eyabya no. 2 | VR nr. 13 | Kagera, Morogoro | Stalk, Roots | Induction of abortion, Used by breastfeeding mother as Galactagogue | Stalk is cut and inserted into the vagina |  | (Millinga et al., 2022; Nikolajsen et al., 2011; Rasch et al., 2014) |
|  | *Clutia abyssinica* Spach. | Umutarishonga (Kinyarwanda) |  | Kagera | Roots | Against habitual abortion | Dried root, together with other plants | Chancre, Inﬂuenza, Dizziness, Ascarifuge, Convulsions, Enlarged spleen and Inﬂuenza | (Ramathal and Ngassapa, 2001) |
|  | *Spirostachys africana* Sond. | Ormatanga,Mharaka |  | Dodoma and Morogoro | Root | Dysmenorrhoea |  | tumour, stomachache | (Hilonga et al., 2019) |
|  | *Phyllanthus sp* | Mgara, Mwepesi | RMK026, RMK038 | Kigoma | Roots/ Leaves | Increases women fertility | Fresh leaf or root decoction is taken orally, or through enema. | Anaemia, and stomach upset, neuropathy, and reduction of body and weight/cholesterols | (Kingo and Maregesi, 2020) |
|  | *Margaritaria discoidea* (Baill.) G. L. Webster | Kasenga | Augustino 109 | Tabora | Roots | Infertility | Oral |  | (Augustino et al., 2011) |
|  | *Oldfieldia dactylophylla* (Oliv.) J. Léonard | Muliwanfwengi | Augustino 149 | Tabora | Roots | Infertility | Oral | Aphrodisiac, hernia, and stomach ache | (Augustino et al., 2011) |
| Fabaceae | *Afzelia quanzensis* Welw. | Maharongome (Makua), Mkongo (Zaramo), Mkola | 1222 1256 | Pwani, Dodoma, Mbeya and Morogoro | Stem bark, Roots | Prevent abortion, Dysmenorrhoea | Powdered and mixed with porridge and taken orally | Conjunctivitis, Snake bite, Aphrodisiac, Pneumonia, Malaria, Stomachache, epilepsy, malaria, and diarrhoea | (Chhabra et al., 1987; Hilonga et al., 2019) |
|  | *Brachystegia boehmii* Taub | Mvomaro (Pare) | 3121 | Morogoro | Roots | Against sterility in females | Decoction of root with that of Msegese | Malaria and Wounds | (Chhabra et al., 1987) |
|  | *Brachystegia spiciformis* Benth. | Myombo (Swahili) | 75 1954 | Pwani | Roots, Leaves | Against menorrhagia | Decoction of roots with infusion of leaves | Bilharzia, Conjuctivitis and pysichriatic cases | (Chhabra et al., 1987) |
|  | *Cassia burttii* Bak. F. | Mkwizingi (Zaramo) | 1681 | DSM | Rootbark | Against Amenorrhoea | Powerded rootbark and decoction of roots added to poridge | Constipation, Hookworm and Ascariasis | (Chhabra et al., 1987) |
|  | *Cassia occidentalis* L. | Kundekunde Mlingajini (Zaramo) Mwambala simba (Zigua) | 555 2823 2896 | Pwani DSM | Root | Againt dysmenorrhoea Against sterility in females | Decoction of root taken orally | Hernia, Enterlalgia, Snake bite, Fever, Kidney trouble and Antibiotic action | (Chhabra et al., 1987) |
|  | *Piliostigma thonningii* (Schumach.) Milne-Redh | Msegese (Swahili),Njolwambogo, Msegese | 375 587 | Pwani, Morogoro | Root, Leaves | Against menorrhagia, Against sterility in females, Women's stomach ache | Decoction of root taken orally | Stomachache, Rectal prolapse, Nausea, Tropical and malignant ulcers | (Abdallah et al., 2007; Augustino and Gillah, 2005; Chhabra et al., 1987) |
|  | *Cassia cfr. singueana* Del. | Mvumba(Digo) | 53 | Tanga | Roots | Galactagogue | Decoction of roots taken orally | Bronchopneumonia, Wounds, Gonorrhoea, Stomach problems, Convulsions, Vinereal diseases, Malaria, Snake bites, Constipation and Heart burn | (Hedberg and Hedberg, 1982) |
|  | *Tamarindus indica* L. | Mkwedu (Makonde) Mkwaju (Swahili) | 155 | Tanga, Pwani | Leaves, Roots | Against Amenorrhoea, Infertility | Decoction of roots taken orally | Vomitting, Hookworm, Lepra, Heart pain, Cough, Fever, Wounds, Antiasmatic, Abcesses, Diarrhoea, and Snake bite | (Abdallah et al., 2007; Hedberg and Hedberg, 1982) |
|  | *Cassia abbreviata* Oliv.subsp. beareana (Holmes) Brenan. | Mwalola (Makonde), Mulundalunda, Mmulimuli, Munzoka, Singwai | 163, Augustino 152, ITM 3708 | Tanga, Tabora, Mbeya, Dodoma, Morogoro | Roots, leaves, Bark, | Against uterine complaints, Infertility, abortion, Dysmenorrhoea | Decoction of roots taken orally | Malaria,stomachache,amoebiasis, sexually transmitted diseases, and diabetes | (Augustino et al., 2011; Hedberg and Hedberg, 1982; Hilonga et al., 2019) |
|  | *Cassia didymobotrya* Fres. | Mwinu (Sambaa) | 182 272 | Tanga | Roots | Against frequent abortion | Decoction of roots taken orally | Mental illines, Purgative, Emetic, Malaria, Headache, Excessive bile, Antidote against general poisoning, Gonorrhoea, Backache in women Measles | (Hedberg and Hedberg, 1982) |
|  | *Cassia mimosoides* L | Orwangwe, Omutoma | PHS nr. 47 | Kagera | Leaves, Stems, Flowers | Induction of abortion | Plant decoction taken orally |  | (Nikolajsen et al., 2011; Rasch et al., 2014) |
|  | *Caesalpinia volkensii* Harms | Mvoro | 3093 |  | Leaves | Relieve abdominal pains during pregnancy | Decoction of leaves taken orally | Malaria | (Chhabra et al., 1984) |
|  | *Abrus precatorious* L. | Kitinutimu (Swahili) Lufambo, Rufumbo, Ufambo (Pare, Zaramo,Zigua), Lufambo, Mwangaruchi, Luvambo (Kipare). | 2801 2827 EA078 49 61 | Pwani, Morogoro, Tanga | Roots, Seeds | Uterine prolapse, Women fertility, Oral contraceptives | Decoction of root taken orally, Powdered seeds | Vomitting, Dysentry, Epilepsy, Stomach aches, Aphrodisia, Convulsions, Abdominal pain, nake bites, Gonorrhea, Sexual impotence, Chest pain, Asthma and Conjuctivitis | (Amri and Kisangau, 2012; Chhabra et al., 1990; Hedberg et al., 1983) |
|  | *Dalbergia melanoxylon* Guill.et Perr. | Gembe (Sukuma), Mpingo (Swahili, Zigua) Poya (Ndengereko) | 103 581 2754 | Pwani, Iringa | Roots | Against uterine prolapse, Infertility | Decoction of root taken orally | Abdominal pain, Hernia, Sexual impotence, Dysuria and Hiccups | (Augustino and Gillah, 2005; Chhabra et al., 1990) |
|  | *Dalbergia vacciniifolia* Vatke | Mgolinyika (Zaramo) | 1717 | Pwani | Roots | Against mastitis | Infusion of leaves mixed with cow's milk and taken orally | Purgative | (Chhabra et al., 1990) |
|  | *Indigofera arrecta* A. Rich | Mkwamba maji (Swahili) Nyanje (Pare) | 3118 | Morogoro | Rootbark | Alleviate labour pains | Decoction of root bark taken orally | Mental illness, Hernia, Colic, Griping, Itching, Gonorrhea, Stomach aches snd Dislocated joints | (Chhabra et al., 1990) |
|  | *Lonchocarpus bussei* Harms | Mfumbili (Zigua), Msabuni (Zaramo), Muwale (Mng’wale), Mnyinga (Digo), Male | 1792 2998 93 221 | Pwani, Tanga, Morogoro | Stem bark, Roots, Leaves, Bark | Against menorrhagia, Against infertility, Galactagogue, Speed up delivery and facilitate extraction of the after birth, Against infertility Improves lactation in women, | Decoction of stem bark, leaves and roots taken orally | Abdominal pain, Hernia, Cardiac palpitations, Bilharzia, Cough, Diuretic, Gonorrhoea, Antibacterial and Palpitation | (Chhabra et al., 1990; Hedberg et al., 1983; Hilonga et al., 2019) |
|  | *Lonchocarpus capassa* Rolfe | Mfumbili (Zigua) | 213 313 | Pwani | Roots | Prevent miscarriage | Infusion of roots is taken orally | Limb pain, Vaginal prolapse, Cold, Hookworms, Convulsions, Mental disorders, Swollen knees, Laxative, Diarrhoea, Leprosy, Snake bites, Antigonoccocal and Antibacterial | (Chhabra et al., 1990) |
|  | *Pericopsis angolensis* (Bak.) van Meeuwen | Mvanga (Hehe) Muwanga (Swahili) Mhagata (Zigua) Mninga (Swahili) Mtumbati (Ndengereko, Pogoro) | 2919, 353, 1972 | Morogoro, Pwani | Roots | Induce abortion Against excessive menstrual bleeding | Root decoction taken orally | Nausea, Diarrhoea Toothache, Stomachache Ascariasis,Schistosomiasis, Abdominal pains, Anemia, Ringworms, Intestinal parasites, Inflamation, Bleeding gums and Antimicrobial | (Chhabra et al., 1990) |
|  | *Cajanus cajan* (L.) Millsp | Mbainisiri (Mbaazi) Digo (Swahili) Entandaigwa, Mubaazi | 37, 289, MJM 3226, Augustino 121 | Tanga, Pwani, Kagera, Tabora | Roots, Leaves, Aerial parts, Seeds | Against stomach ache by women suspecting to be/ during pregnant, Treatment of nausea during pregnancy, Placenta expulsion, antiabortion, infertility, foetus disposition, labour progression | A decoction of roots and leaves taken orally | Wounds, Scalds, Toothache, Gonorrhoea, Bad vision, Heart diseases, Stomach troubles, Gonorrhoea, Tooth aches, Posoning, Nausea, Swelling of legs | (Augustino et al., 2011; Chhabra et al., 1990; Hedberg et al., 1983; Moshi et al., 2012) |
|  | *Senna occidentalis* (L.) Link /*Cassia abbreviata* Oliv | Mkundekunde (Swahili) Mlundalunda (Swahili)Olsingwai (Masai), Mwitanjoka |  | DSM | Leaves | Abortion, Dysmenorrhoea |  | Hypertension, HIV/AIDS, Hernia | (Hilonga et al., 2019; Lindh, 2015) |
|  | *Acacia sp.* | Kiloriti (Masai) |  | DSM |  | Menstruation |  |  | (Lindh, 2015) |
|  | *Hymenaea verrucosa* Gaertn. | Mkumbi (Swahili) Olbukoi (Masai) |  | DSM |  | Milk production Pregnancy |  |  | (Lindh, 2015) |
|  | *Abrus sp* | Ufjambo (Swahili) Rufambu (Kwere) |  | DSM |  | Right before birth |  |  | (Lindh, 2015) |
|  | *Trigonella foenumgraecum* L. | Uwatu (Swahili) |  | DSM |  | Right after birth |  |  | (Lindh, 2015) |
|  | *Crotalaria cf. caudata* Welw. ex Baker | Bulebasubhugu (Jita) Kaninagu (Sukuma) | SMM-BD17 | Mara | Leaf | Against threatened miscarriage | Leaf decoction is taken orally | Gonorrhea Skin disease Insanity | (Maregesi et al., 2007b) |
|  | *Albizia anthelmintica* (A. Rich.) Brongn. | Mfleta, Mfuleta (Zigua), Mukutani,Mfuleta | 434 1026 | Pwani, Dodoma, Arusha, Morogoro, Mbeya, Njombe | Roots | Against female sterility, Sexual stimulant in females, Dysmenorrhoea | Decoction of root taken orally | Stomach aches, Impotence, Epilepsy, Gonorrhoea, Syphilis, Psychiatric problems, Chest pains, Aphrodisiac, Burns, Antimalarial, Fever, Anthelmintic, Nervous complaints, bile/jaundice,worms, Libido disorder, and kidneyproblems, | (Chhabra et al., 1990; Hilonga et al., 2019) |
|  | *Albizia harveyi* Fourn. | Mhonya (Zigua) Msisimisi (Swahili,Zigua) | 180 210 1833 | Pwani | Roots | Against abdominal pain in pregnant women Against infertility Prevent accidental abortion Promote conception | Infusion of root is taken orally | Epilepsy cystitis Vomiting Intestinal troubles Antigonococcal activity | (Chhabra et al., 1990) |
|  | *Dichrostachys cinerea* (L) Wight et Arn. Subsp. Africana Brenan et Brummiitt | Kikulagembe (Matumbi), Mkulagembe (Swahili), Mutundulu, Mgegele, Mtogo | 1680, 1953, Augustino 187 | Pwani, DSM, Tabora, Iringa, Morogoro | Roots, leaves, Bark | Against menorrhagia, Against threatened abortion, Infertility, menstrual disorder | Decoction of root taken orally | Burning abdominal pains Blood diarrhea, Pneumonia, Persistent cough, Asthma, Hernia, Malaria, Snake bites, Joints, Bone diseases, Heart pains, Wound healing, Pneumonia, Scorpion bites, Chest complaints Leprosy, Syphilis, Polmunary tuberculosis, Mental diseases wounds, epilepsy, rituals, , rectal prolapse, stomach ache, diarrhoea, dizziness | (Augustino et al., 2011; Augustino and Gillah, 2005; Chhabra et al., 1990) |
|  | *Entada abyssinica* Steud. Ex A. Rich | Mfufuma simba (Zigua), Mufutwambula, Ngemwambula | 3132, Augustino 128 | Morogoro, Tabora | Roots, leaves, Bark | Against menorrhagia, Infertility | Decoction of roots together with that of mtogo is taken orally | Kidney problems, Rheumatism, cough, catarrh, fever, Sores, Malaria, Bronchial engorgement Abdominal troubles | (Augustino et al., 2011; Chhabra et al., 1990) |
|  | *Entada stuhlmannii* (Taub.) Hams | Mmehagona (Zaramo) | 403 | Pwani | Roots | Lactagogue | Infusion of root is taken orally | Stomach pain Aphrodisiac | (Chhabra et al., 1990) |
|  | *Vachellia abyssinica* (Hochst. ex Benth.) Kyal. & Boatwr (Acacia abyssinica Hochst. ex Benth) | Umunyinya (Kinyarwanda) |  | Kagera | Leaves | Treat mastitis. | Infusion of the leaves taken orally |  | (Ramathal and Ngassapa, 2001) |
|  | *Vachellia kirkii* (Oliv.) Kyal. & Boatwr (*Acacia kirkii* Oliv.) | Mkongowe (Pare, Vidunda) | 2941 3068 | Morogoro Moshi | Roots | Against female sterility | Roots are cooked with chicken meat and soup taken orally | Stomach disease Pains in the back Antibacterial | (Chhabra et al., 1989) |
|  | *Senegalia pentagona* (Schumach.) Kyal. & Boatwr(*Acacia pentagona* (Schumach.) Hook.f. | Magowera (Zaramo) | 3136 | Pwani | Roots | Againt amenorrhoea | Decoction of root taken orally |  | (Chhabra et al., 1989) |
|  | *Senegalia polyacantha* (Willd.) Seigler & Ebinger (*Acacia polyacantha* Willd.) | Mgunga (Zigua) | 1428 | Pwani | Roots | Against vaginal occlusion | Decoction of root taken orally | Stomach pains Whooping cough Asthma Sores Snake bites Malaria | (Nikolajsen et al., 2011) |
|  | *Vachellia robusta*(Burch.) Kyal. & Boatwr. (*Acacia robusta* Burch) | Mkongowe (Pare, Zigua, Swahili) Muhemba (Zigua) Mwangang'ombe (Zaramo) Mwanzi (Haya) | 1776 2132 3138 | Pwani | Roots | Against dysmenorrhia Female sterility | Decoction of root taken orally | Swellings Schistosomiasis Gonorrhoea Abdominal pains Antibacterial | (Chhabra et al., 1990) |
|  | *Vachellia tortilis* (Forssk.) Galasso & Banfi (*Acacia tortilis* (Forsk.) Hyne) | Mkongowe (Zigua), Ng’ale (Sukuma) | 231, SMM-BD29 | Pwani, Njombe | Roots | Against irregular menses, Corrections of irregular menses | Decoction of root taken orally | Mouth infections Dental problems Malaria | (Chhabra et al., 1990; Maregesi et al., 2007b) |
|  | *Senegalia nigrescens* (Oliv.) P.J.H.Hurter (*Acacia nigrescens* Oliv.) | Mkambala (Zigua) | 1750 | Pwani | Roots | Increase libido | Root decoction taken with chicken soup |  | (Chhabra et al., 1989) |
|  | *Senegalia mellifera* (Vahl) Seigler & Ebinger (*Acacia mellifera* (Vahl) Benth.) | Mnoa (Pare) Msasa (Swahili) | 39 3084 | Pwani Moshi | Roots, Stem bark | Against pain during menstruation Against infertility Against mastitis | Decoction of root taken orally Stem bark applied on breast | Impotence, Diarrhoea Rectal prolapse, Aphrodisiac, Cancer, Stomach troubles, Pneumonia, Malaria, Syphilis and Antibacterial | (Chhabra et al., 1989) |
|  | Se*negalia brevispica* (Harms) Seigler & Ebinger (*Acacia brevispica* ) | Msewa (Zigua), Bigeye (Jita), Ikeye,Girgiri, Lugeye (Sukuma), Mdunga | 28, SMM-BD19 | Pwani, Mara, Morogoro | Roots, bark | Against female sterility, Against female infertility, Dysmenorrhoea, Menorrhagia | Roots are cooked with chicken meat and soup taken orally; Decoction; oral | Cough, Intestinal worms, Snake bite, Aphrodisiac, Antihelminthic, Abcesses, Against evils pirits | (Abdallah et al., 2007; Chhabra et al., 1989; Hilonga et al., 2019; Maregesi et al., 2007b) |
|  | *Sesbania sesban* (L) M.S | Zuzuma (Sukuma) | SMM-BD49 | Mara | Seeds | Against excessive mensentral haemorrhage |  | Venereal diseases, Throat sore, Gonorrhoe, Syphilis, Yaws, Children convulsions, Dizziness, Antihelminth and Scorpion bite | (Maregesi et al., 2007b) |
|  | *Desmodium barbatum* (L.) Benth | Omubabazi, Muarobaini | VR nr. 1 | Kagera | Leaves, Stems | Induction of abortion | Handful of plant is chewed |  | (Nikolajsen et al., 2011; Rasch et al., 2014) |
|  | *Macrotyloma axillare* (E. Mey.) Verdc | Eyabya, Eyabia | PHS nr. 44 | Kagera | Leaves | Induction of abortion | Decoction of leaves taken orally |  | (Nikolajsen et al., 2011; Rasch et al., 2014) |
|  | *Crotalaria sp* (Papilionaceae) | Akayogera (Kinyarwanda) |  | Kagera | Leaves | To ease childbirth Treatment of pain after delivery | Infusion of the leaves taken orally |  | (Ramathal and Ngassapa, 2001) |
|  | *Arachis hypogaea* L |  |  | Morogoro |  | Used by breastfeeding mother as Galactagogue |  |  | (Millinga et al., 2022) |
|  | *Julbernardia globiflora* (Benth.) Troupin | Kilepori |  | Morogoro, Mbeya | Root | infertility |  | Spinalcordpain,hernia, jointpain,stomachache | (Hilonga et al., 2019) |
|  | *Albizia lebbeck* (L.) Benth. | Mlongelonge, Myenjeyenje |  | DSM | Flower, Leaves | Dysmenorrhoea |  | malaria,diarrhoea | (Hilonga et al., 2019) |
|  | *Senna alata* (L.) Roxb. | Njorwambogo |  | Morogoro | Leaves | Dysmenorrhoea |  |  | (Hilonga et al., 2019) |
|  | *Vachellia seyal* (Delile) P.J.H. Hurter | Elerai,Naju,Oltepesi |  | Njombe, Dodoma. Morogoro, Mbeya | Root | Dysmenorrhoea |  | Stomachache,malaria,hernia | (Hilonga et al., 2019) |
|  | *Acacia mellifera* (Vahl) Benth. | Mulugala | Augustino 150 | Tabora | Roots, leaves, Bark | Menstrual disorder | Oral | Stomach ache, diarrhoea, anaemia | (Shangali et al., 2008) |
|  | *Acacia nigrescens* Oliv. | Kagowole | Augustino 106 | Tabora | Roots | Infertility | Oral, Bath | foetus disposition, stomach ache, lucky | (Augustino et al., 2011) |
|  | *Mundulea sericea* (Willd.) A.Chev. | Mutandala | Augustino 182, ITM 3710 | Tabora | Roots, bark | Antiabortion | Oral, nasal, massage | wounds, aphrodisiac, hernia, bilharzia, epilepsy, stomach ache | (Augustino et al., 2011) |
|  | *Pterocarpus tinctorius* Welw. | Mukurungu | Augustino 147, ITM 3712 | Tabora | Roots, leaves, Bark | Antiabortion | Oral, massage | Anaemia, diarrhoea, wounds, stomach ache, eyes ache, snakebite | (Augustino et al., 2011) |
|  | *Acacia tortilis* (Forssk.) Hayne Syn: *Vachellia tortilis* (Forssk.) Galasso & Banfi | Mngokowe |  | Morogoro | Roots | Late labour pains, Infertility, Women's stomach ache |  | Gonorrhea /Stroke /Convulsion | (Augustino and Gillah, 2005) |
|  | *Acacia albida* Delile Syn of *Faidherbia albida* (Delile) A. Chev. | Mkongolo, Mpogolo |  | Morogoro, Iringa | Leaves, Roots | Pregnancy poison/ complication, Infertility |  |  | (Augustino and Gillah, 2005) |
|  | *Acacia polyacantha* Willd. Syn of *Senegalia polyacantha* (Willd.) Seigler & Ebinger | Mtandula, Muwindi |  | Morogoro | Roots | Late labour pains, Infertility |  |  | (Augustino and Gillah, 2005) |
|  | *Pterocarpus angolensis* DC | Mhagata |  | Morogoro | Roots | Women abdominal pains after delivery |  |  | (Augustino and Gillah, 2005) |
| Flacourtiaceae | *Trimeria grandifolia* (Hochst.) Warb. | Mdagha | 3099 |  | Root | Against dysmenorrhoea | Decoction of root taken orally | Mental illness Swollen scrotum Gonorrhoea Abdominal troubles | (Chhabra et al., 1984) |
|  | *Scolopia stolzii* Gilg | Mgogola |  | Iringa | Roots | Conception | Boil then drink |  | (Kitula, 2007) |
| Guttiferae/ Clusiaceae | *Harungana madagascariensis* Lam. ex Poir. | Mvavata, Umushayishayi (Kinyarwanda) |  | Morogoro, Iringa, Kagera | Roots, Leaves | Infertility, Against amenorrhoea | A decoction from the roots is taken orally for seven days; Infusion of the leaves taken orally | Haemorrhages, Diarrhoea, Gonorhoea, Sore throat, Fevers, Worm infestations | (Ramathal and Ngassapa, 2001; Shangali et al., 2008) |
| Hymenocardiaceae | *Hymenocardia acida* Tul. | Mupala | Augustino 166 | Tabora | Roots, Leaves | Infertility | Oral, anal, nasal | Epilepsy, rectal prolapse, hernia, stomach ache | (Augustino et al., 2011) |
| Lamiaceae | *Plectranthus barbatus*.  Syn; *Plectranthus kilimandschari* Gurke | Makoroma (Jita) | SMM-BD47 | Mara | Leaf | Against dysmenorrhea | Leaf infusion is taken orally | Chest pain, Cough, Psychiatric problems Phagedenic ulcers | (Maregesi et al., 2007b) |
|  | *Coleus barbatus* var. grandis  Syn; *Coleus kilimandschari* Gurke | Igicunshu (Kinyarwanda) |  | Kagera | Leaves | Abortifacient Used by expectant mothers to lighten labour | Infusion of the leaves taken orally | Stomach pains, Heartburn, Mouth foaming | (Ramathal and Ngassapa, 2001) |
|  | *Leonotis nepetifolia* R. Br. | Igicumucumu (Kinyarwanda) |  | Kagera | Whole plant | Against dysmenorrhoea | Decoction of the whole plant taken orally | Antiemetic, Tapeworms, Rheumatism, Syphilitic ulcers, Skin diseases, Antiasthmatic, Urinary remedy, and Typhoid | (Ramathal and Ngassapa, 2001) |
|  | *Ocimum urticifolium* Roth | Umwenya (Kinyarwanda) |  | Kagera | Whole plant | Stop bleeding after abortion. | Decoction of the whole plant taken orally |  | (Ramathal and Ngassapa, 2001) |
|  | *Ocimum suave* Willd | Akashwagara, Lwenyi, | VR nr. 3 | Kagera, Iringa | Leaves | Induction of abortion, Backache after delivery | Decoction of leaves taken orally; Pound mix with sheep tail fat then Insert to the anus |  | (Kitula, 2007; Nikolajsen et al., 2011; Rasch et al., 2014) |
|  | *Orthosiphon suffrutescens* (Schumach.) J. K. Morton | Olemorani |  | Morogoro | Leaves | Infertility health complaints |  |  | (Hilonga et al., 2019) |
|  | *Premna senensis* Klotzsch | Mununhwanhala | Augustino 162 | Tabora | Roots, leaves | Infertility | Oral, nasal, massage, bath, external | Epilepsy, body pains/weakness, stomach ache, hernia, aphrodisiac, abscess, rituals | (Augustino et al., 2011) |
|  | *Vitex mombassae* Vatke | Mutalali, Musungwi | Augustino 181 | Tabora | Roots, leaves | Infertility | Oral, massage | Body rashes, stomach ache, diabetes, diarrhoea, snakebite | (Augustino et al., 2011) |
|  | *Vitex doniana* Sweet | Mfuru |  | Morogoro | Roots | Women stomach ache |  |  | (Augustino and Gillah, 2005) |
|  | *Vitex mombassae* Vatke | Sungwi |  | Morogoro | Roots | Infertility |  |  | (Augustino and Gillah, 2005) |
| Lauraceae | *Cinnamomum verum* J. Presl |  |  | Morogoro |  | Used by breastfeeding mother as Galactagogue |  |  | (Millinga et al., 2022) |
|  | *Ocotea usambarensis* Engl. Syn of *Kuloa usambarensis* (Engl.) Trofimov & Rohwer | Mseri |  | Morogoro | Barks | Women's stomach ache, Infertility |  | Love, Infants complications, Anaemia | (Augustino and Gillah, 2005) |
| Linaceae | *Hugonia arborescens* Mildbr. | Nyambu (Zigua) |  | DSM |  | Miscariage |  |  | (Lindh, 2015) |
| Loganiaceae | *Nuxia floribunda* Benth. | Mngogo |  | Iringa | Roots | Irregular menstruation period | Pound, boil then drink |  | (Kitula, 2007) |
|  | *Strychnos potatorum* | Mshindwi | RMK039 | Kigoma | Roots/ Leaves | Dilates cervix in pregnant women. | Clean grounded fresh leaf or root paste is applied at the vagina near term |  | (Kingo and Maregesi, 2020) |
|  | *Strychnos spinosa* Lam. | Ngodakalomo |  | Morogoro | bark, root | Dysmenorrhoea |  |  | (Hilonga et al., 2019) |
| Malvaceae | *Grewia fallax* K. Schum | Mlawa | 3108 |  | Root | Prevent abortion | Decoction of root taken orally | Snake bite | (Chhabra et al., 1984) |
|  | *Hibiscus sabdariffa* L. | Rozela (Swahili)/Damdam (Swahili) Mdamu damu | 5036 | DSM, Pwani | Leaves, Calyces | Anaemia |  |  | (Lindh, 2015; Peter et al., 2014) |
|  | *Triumfetta microphylla* Wight & Arn | Kaitampunu |  | Kagera |  | Induction of abortion |  |  | (Rasch et al., 2014) |
|  | *Grewia bicolor* Juss. | Mukoma | Augustino 140 | Tabora | Roots | Infertility | Oral | Anaemia | (Augustino et al., 2011) |
|  | *Grewia conocarpoides* Burret | Mudati | Augustino 125 | Tabora | Roots | Infertility | Oral |  | (Augustino et al., 2011) |
|  | *Sterculia africana* (Lour.) Fiori | Muhozya | Augustino 136 | Tabora | Roots, bark | Infertility | Oral, bath, external | Pain relief, anaemia, mental disorder, convulsion, lucky, rituals | (Augustino et al., 2011) |
|  | *Thespesia garckeana* F. Hoffm. | Mutobo | Augustino 185 | Tabora | Roots | Labour progression | Not specified |  | (Augustino et al., 2011) |
|  | *Grewia similis* K. Schum. | Mkole |  | Pwani | Roots | Dysmenorrhoea | Decoction; oral |  | (Abdallah et al., 2007) |
|  | *Thespesia danis* Oliv. | Mmoyomoyo |  | Pwani | Roots | Infertility; Menorrhagia | Decoction; oral |  | (Abdallah et al., 2007) |
|  | *Adansonia digitata* L. | Mbuyu (Swahili) | 130 | Tanga, Morogoro, Iringa | Stembark, Flowers, Barks | Against menorrhagia, Women stomach ache, Infertility | Stembark decoction taken orally | Less pronounced vaginal bleeding, Lassitude, Malaria, Toothache, Body pains, Diaphoretic, Anemia, Tonic, Febrifuge, Rachitis, Expectorant, Astringent and Prophylactic against fevers | (Augustino and Gillah, 2005; Hedberg and Hedberg, 1982) |
| Melastomataceae | *Sphaerogyne latifolia* Naudin | Omubabazi | VR nr. 11 | Kagera | Leaves | Induction of abortion | Decoction of leaves taken orally |  | (Nikolajsen et al., 2011; Rasch et al., 2014) |
|  | *Dissotis rotundifolia* (Sm.) Triana Syn of *Heterotis rotundifolia* (Sm.) Jacq.-Fél. | Ling'eng'ena |  | Njombe | Leaves | Stop abortion |  | Rheumatism, painful swellings, Stomache, Diarrhoe, Dysentery, Cough, Conjunctivitis, Circulatory problems and Venereal diseases | (Kayombo, 2016) |
| Meliaceae | *Cedrella odorata* L. | Mwerezi | EA079 | Morogoro | Leaves/bark | Menstrual cycle and Women fertility; | Infusion taken orally |  | (Amri and Kisangau, 2012) |
|  | *Azadirachta indica* A. Juss | Muarobaini (Swahili), Kikikarabo | VR nr 4 | DSM, Kagera, Mwanza, Morogoro | Leaves | Induction of abortion, Used by breastfeeding mother as Galactagogue | Decoction of leaves taken orally | HIV/AIDS | (Dika et al., 2017; Lindh, 2015; Millinga et al., 2022; Nikolajsen et al., 2011; Rasch et al., 2014) |
|  | *Turraea fischeri* Gürke | Ningiwe, Muningiwe | Augustino 203 | Tabora | Roots | Infertility | Oral, massage | Stomach ache, headache | (Augustino et al., 2011) |
| Mimosaceae | *Albizia anthelmintica* (A.Rich.) Brongn. | Mfulete |  | Morogoro, Iringa | Roots | Infertility |  |  | (Augustino and Gillah, 2005) |
| Moraceae | *Ficus sycomorus* L. | Mkuyu, Mkunju (Kwere) Engiloilo (Masai) Mkuso (Zigua) | EA067 | Morogoro, DSM | Bark | Menstrual cycle, Women fertility; Infertility | Infusion taken orally |  | (Amri and Kisangau, 2012; Lindh, 2015) |
|  | *Ficus capensis* Thunb | Mkuyu (Swahili) | 2953 | Morogoro | Roots | Treatment of breast against mastitis Galactagogue | Infusion of root is taken orally to wash breast | Hernia, Convulsions, Psychriatic problems, Toothaches, Septic conjunctivitis, Stomach upsets, Rachitiis and Antibacterial | (Chhabra et al., 1990) |
|  | *Ficus natalensis* Hochst | Mlumba (Luguru) | 1133 | Morogoro | Roots | Galactagogue | Decoction of root taken orally | Malaria, Snake bite, Influenza, Colic, Syphlis and Skin conditions | (Chhabra et al., 1990) |
|  | *Ficus thonningii* Blume | Umuvumu (Kinyarwanda), Omutoma, Omushangati |  | Kagera | Roots | To stimulate lactation, Induction of abortion | Decoction of the whole plant, root taken orally, | Poisoning | (Nikolajsen et al., 2011; Ramathal and Ngassapa, 2001; Rasch et al., 2014) |
|  | *Ficus sur* Forssk. | Ng'aboli,Twaligana, Msombe |  | Morogoro, Mbeya, DSM, Iringa | Leaves | Milk production, Infertility |  | diarrhoea | (Augustino and Gillah, 2005; Hilonga et al., 2019) |
|  | *Ficus sp.* |  |  | Iringa | Barks | Boil on breast during breastfeeding | Pound. Boil, smear on breast |  | (Kitula, 2007) |
| Moringaceae | *Moringa oleifera* Lam. | Moringa, Omolongélóngé/ emelongélóngé, Mlongelonge |  | Mwanza, Mara, Morogoro | Leaves, Seeds, Barks | Nutrition supplement during pregnancy, To stimulate milk for breastfeeding | Powdered leaves mixed with yogurt; Boil in water chewing seeds, pound, dry and grind – to be taken orally in the tea/milk | Gastric or peptic ulcers, Asthma | (Augustino and Gillah, 2005; Bisanz et al., 2015; Charwi et al., 2023) |
| Musaceae | *Ensete ventricosum* | Linyinyimbi |  | Morogoro, Iringa | Leaves | Prolonged menstruation | Juice from leaves in water solution is taken orally or fresh leaves are chewed and juice swallowed |  | (Shangali et al., 2008) |
| Myrsinaceae | *Maesa lanceolota* Forssk | Mhenyi, Mguti |  | Morogoro, Iringa | Barks, Roots | Boil on breast during breastfeeding, Infertility | Pound. Boil, smear on breast; A decoction from the roots is taken orally for seven days |  | (Kitula, 2007; Shangali et al., 2008) |
|  | *Embelia schimperi* Vatke | Mnyanyinyanyi |  | Iringa | Leaves | Women stomach ache |  |  | (Augustino and Gillah, 2005) |
| Myrtaceae | *Psidium guajava* L | Mpera (Swahili) |  | Njombe | Leaves | Treatment of uterine haemorrhage Wash for uterine and vaginal problems Promotes menstruation | Decoction of leaves taken orally | Fevers, Diabetes, Epilepsy, Worms, Spasms, Swollenness of the legs and other parts of the body, Chronic diarrhea, Dysentery, Gastroenteritis, Inflammation of the kidneys, Astringent, and Ulcers | (Kayombo, 2016) |
|  | *Syzygium aromaticum* (L.) |  |  | Morogoro |  | Used by breastfeeding mother as Galactagogue |  |  | (Millinga et al., 2022) |
| Ochnaceae | *Brackenridgea zanguebarica* Oliv | Mkatakwa (Zigua) Mlukama (Makua) | 1044 | Pwani | Roots | Against dysmenorrhoea Against amenorrhoea | Decoction of root taken orally | Anemia, Rectal prolapse, Oedema, Intestinal worms, Conjuctivitis, Scabies, Wounds, Aphrodisiac, Snake bites and Antithrombotic | (Chhabra et al., 1990) |
| Olacaceae | *Ximenia americana* L. | Lusasalwake (Zigua), Mtundwa (Sukuma), Ngomai | 2457 | Dodoma, Mbeya and Morogoro | Roots | Female complaints during pregnancy; Dysmenorrhoea, irregular menstrual period | Decoction of root taken orally | Convulsions, Diarrhoea, Ulcers, Craw-craw, Ring worm, Venereal diseases, Intestinal gas, Homeostatic, hormones, and Libido disorder | (Chhabra et al., 1990; Hilonga et al., 2019) |
|  | *Ximenia caffra* Sond. Var. caffra | Mpingi (Swahili), Mtundutwa (Pare), Mtundwi (Zigua), Mtundwa, Tundwa | 1356 3062 | Pwani, Morogoro, Iringa, Mbeya | Roots, Leaves | Against irregular menses, Against uterine prolapse, Against lower abdominal pains in women, Against female sterility, Dysmenorrhoea | Decoction of root taken orally | Rheumatism, Ulcers, Cancer, Intestinal worms, Hernia, Plague, Mental disorders, Bloody diarrhoea, Stomach aches Malaria, Bilharzia, Hookworms, Chest pains, Syphlis, Trachoma, | (Augustino and Gillah, 2005; Chhabra et al., 1990; Hilonga et al., 2019) |
|  | *Ximenia sp.* | Mpingi (Swahili) Engamai (Masai) |  | DSM |  | Menstruation Pregnancy Right after birth |  |  | (Lindh, 2015) |
|  | *Jasminum fluminense* Vell. | Chingula |  | DSM | Leaves | Infertility |  |  | (Hilonga et al., 2019) |
|  | *Strombosia scheffleri* | Mkongotsa |  | Morogoro, Iringa | Roots | Infertility | A decoction from the roots is taken orally for seven days |  | (Shangali et al., 2008) |
| Oleaceae | *Jasminum ﬂuminense* Vell | Binyafwira (Jita), Kihagadume | SMM-BD15 | Mara, Morogoro, Iringa | Leaf, Roots | To treat female infertility | Macerated leaves taken orally; A decoction from the roots is taken orally for seven days | Cellulites, Abscess, Snake bite, Uvulitis, Antihelminthic, Antirheumatic and Antidiarrhoea | (Maregesi et al., 2007b; Shangali et al., 2008) |
| Onagraceae | *Ludwigia abyssinica* A. Rich | Umuzingangore (Kinyarwanda) |  | Kagera | Leaves | To prevent abortion | Infusion of the leaves taken orally |  | (Ramathal and Ngassapa, 2001) |
| Opiliaceae | *Opilia celtidifolia* (Guill.et Perr.) Endl. Ex Walp. | Mkandekande (Swahili) Mwevumbulo (Zigua) | 1676 | DSM | Roots | Uterine stimulant | Decoction of root taken orally | Abdominal pains, Ear aches, Mental illness, Headaches, Fever, Influenza and Diuretic | (Chhabra et al., 1990) |
| Oxalidaceae | *Biophytum helenae* Buscal & Muschi | Orwangwe |  | Kagera |  | Induction of abortion |  |  | (Rasch et al., 2014) |
| Passifloraceae | *Adenia gummifera* (Harv.) Harms | Gore (Swahili, Zaramo) | 385 | DSM | Roots | Against female sterility | Root decoction taken orally | Cholera, Stomachache, Dysentry, Waist and hip pain, Colic, Emetic, Scabies, Hydrocele, Gonorrhoea, Diarrhoea, Anemia, Malaria and Leprosy | (Chhabra et al., 1991) |
|  | *Adenia racemosa* de Wilde | Gole (Pale) | 3113 | Kilimanjaro | Stembark | Against menorrhagia | Stembark decoction taken orally | Mental illines | (Chhabra et al., 1991) |
| Phyllanthaceae | *Phyllanthus reticulatus* Poir. | Mkwambe-mazi | 412 |  | Rootbark | Against sterility in females | Decoction taken orally | Gonorrhea Purgative Hookworms Sores Burns Diuretic Alterative Anemis Intestinal hemorrhage | (Chhabra et al., 1984) |
|  | *Pseudolachnostylis maprouneifolia* Pax | Mutungulu |  | Pwani | Roots | Infertility; Menorrhagia | Decoction; oral |  | (Abdallah et al., 2007) |
|  | *Bridelia micrantha* (Hochst.) Baill. | Mwitsa |  | Iringa | Roots, Leaves | Women's stomach ache, Infertility |  | Tuberculosis, Convulsion | (Augustino and Gillah, 2005) |
| Piperaceae | *Piper nigrum* L |  |  | Morogoro |  | Used by breastfeeding mother as Galactagogue |  |  | (Millinga et al., 2022) |
| Poaceae | *Pennisetum purpureum* Schumach. | Isumbu | Augustino 104 | Tabora | Stem | Infertility | Oral |  | (Augustino et al., 2011) |
|  | *Cymbopogon citratus* Stapf |  |  | Morogoro |  | Used by breastfeeding mother as Galactagogue |  |  | (Millinga et al., 2022) |
| Polygalaceae | *Securidaca longepedunculata* Fresen | Mbazo (Zigua), Nengonengo, Mteyu, Mteyo, Nengonengo | 119, 265, Augustino 120 | Tanga, Morogoro, Tabora | Roots, leaves | Against dysmenorrhoea, To hasten birth, Infertility, placenta expulsion | Infusion of roots taken orally | Gonorrhoea, Heart pain, Headache, Oedema, Purgative, Disorders of genital organs, Astringent, Chest complaints, Rheumatism Gum boil, Nose bleeding, Venereal disease, Epilepsy, and aphrodisiac | (Augustino et al., 2011; Augustino and Gillah, 2005; Hedberg et al., 1983; Hilonga et al., 2019) |
| Polygonaceae | Oxygonum sinuatum (Hochst. & Steud.ex Meisn.) Damm. | Kachumitambogo |  | DSM | Root | Dysmenorrhoea |  | malaria,urinarytract infection,gonorrhoea | (Hilonga et al., 2019) |
|  | *Oxygonum stuhlmannii* Dammer | Mbigili (Swahili) | 3144 | DSM | Whole plant | Against menstrual disorders | Whole plant decoction taken orally |  | (Chhabra et al., 1991) |
|  | *Rumex usambarensis* (Engl, ex Dammer) Dammer. | Nyanywa, Ng’ambu (Sambaa), Umufumbegeshi (Kinyarwanda) | 169 | Tanga, Kagera | Leaves | Against stomach pains during pregnacy, To treat mastitis and abortion | Infusion of leaves taken orally, Leaves are crushed, boiled and applied | Bilharzia Cough Stomach pains Costipation Stomach eruptions Smallpox | (Hedberg et al., 1983; Ramathal and Ngassapa, 2001) |
| Primulaceae | *Lysimachia ruhmeriana* Vatke | Umuyobora (Kinyarwanda) |  | Kagera | Leaves | For expulsion of placenta | Decoction of the leaves taken orally |  | (Ramathal and Ngassapa, 2001) |
|  | *Maesa lanceolata* Forssk. | Mguti |  | Iringa | Roots, Leaves | Infertility |  | Urine Tract infection, Dysentery, Body rashes | (Augustino and Gillah, 2005) |
| Ranunculaceae | *Clematis brachiata* Thunb | Tambariko (Pale) | 3106 | Kilimanjaro | Leaves | Relieving abdominal disorders of pregnant women | Fresh leaves crushed and juice taken orally, Infusion of leaves is taken orally | Syphillis, Cough, Trush, Headache, Abdominal disorders, Chest complaints, Malaria, Cold, Snake bites, and Antibacterial | (Chhabra et al., 1991, 1984) |
| Rhamnaceae | *Scutia myrtina* (Burm. f.) Kurz. | Msiluga (Ziguwa) | 195 | Tanga | Leaves | To hasten parturition of both child and placenta | Leaf paste used as an ointment | Gonorrhoea Bilharzia Intestinal worms | (Hedberg et al., 1983) |
|  | *Ziziphus mucronata* Willd. ssp. mucronata. | Mgagawe (Zigua), Mugugunu | 233, Augustino 131 | Tanga, Tabora | Roots, bark | Prevent abortion, Foetus disposition | Decoction of roots taken orally | Asthma, Snake bite, Dysentry, Glandular swellings, Lumbago, Scrofulous swellings, Pain, Gonorrhoea, Swelling, Diuretic, Mental disorders, Rheumatism, Urine incontinence, Aphrodisiac, stomach ache, chest pains, and hypertension | (Augustino et al., 2011; Hedberg et al., 1983) |
|  | *Rhamnus mucronata* Schltdl. Syn of *Frangula mucronata* (Schltdl.) Grubov | Kihanga, Kihaga |  | Iringa | Roots | Conception | Boil then drink |  | (Kitula, 2007) |
|  | *Rhamnus prionoides* L’Hér. | Likamanda |  | Iringa | Leaves | Milk production |  |  | (Augustino and Gillah, 2005) |
| Rosaceae | *Rubus pinnatus* Willd | Lufifi | EA093 | Morogoro | leaves | Menstrual cycle |  |  | (Amri and Kisangau, 2012) |
|  | *Hagenia abyssinica* (Bruce) J.F.Gmel. | Mluziluzi |  | Morogoro | Roots | Infertility |  |  | (Augustino and Gillah, 2005) |
| Rubiaceae | *Vangueria infausta* Burch. | Mviru, Mviru (Digo), Msada, Msada, Mzambarau | EA084, 70 96 246 | Morogoro, Tanga, Njombe, Iringa | Seeds, roots, Whole plant, Barks | Against menstrual problems, Against menstrual and uterine problems, Infertility | Infusion taken orally, Decoction of root taken orally | Swollen stomach, Hernia, Cough, Chest complaints, Malaria, Fever, Pneumonia, Roundworms, Purgative, Dental pain, Vomiting, AIDS , Gonorrhoea, Stomach disorders, Diarrhoea , Wounds , Pneumonia, Purgatives, Toothache, Ring worms, Genital swellings plasmodial, Infants complications, and Bewitchment | (Amri and Kisangau, 2012; Augustino and Gillah, 2005; Hedberg et al., 1983; Kayombo, 2016) |
|  | *Agathisanthemum bojeri* Klotzsch | Mkwambe (Zigua) Mwima, Ngobolele (Zaramo)Uneke (Ndengereko) | 1621 2104 | Pwani | Roots | Against retained placenta | Root decoction taken orally | Waist pains, Abdominal pains, Intestinal worm, Fever, Communicable diseases, Eye Pain, Diarrhoea, Sore throat, Toothaches, Snake bites, Cough, and Chest Problems | (Chhabra et al., 1991) |
|  | *Catunaregam nilotica* (Stapf) Tirvengadum | Mdasha, Mtongangombe (Zigua) Mpigi, Mpirupiru (Ndengereko) Mtutuma (Zaramo) Mwachanguku (Sukuma) | 223 319 1951 | Pwani | Roots | Against menorrhagia Induce labour Against delayed pregnancy Female infertility | Root decoction taken orally | Abdominal pains, Fevee, Impotency, Mental break down, Convulsions, Emetic, Nervous problems, Asthma, Loss of appetite, Rectal prolapse, Hernia, and Snake bites | (Chhabra et al., 1991) |
|  | *Crossopteryx febrifuga* (G.Don) Benth | Nakapwendo (Makonde), Kumbwambizo (Sukuma), Mhotaponzi | 3119, SMM-BD11 | Pwani, Mara, Iringa | Stembark, Roots | Abortifacient, Used cure female infertility | Dried powdered roots and stembark are mixed with porridge, Decoction of roots taken orally | Constipation, Asthma, Tuberculosis , Hookworm, Veneral disease, Syphilitic ulcer, Conjuctivitis, Cough, Stomachache, Hookworm, Veneral diseases, and Hookworms | (Augustino and Gillah, 2005; Chhabra et al., 1991; Hedberg et al., 1983; Maregesi et al., 2007a) |
|  | *Lasianthus pedunculatus* E.A. Bruce | Mkera (Hehe) | 2928 | Morogoro | Roots | Against female sterility | Root decoction taken orally | Antibacterial | (Chhabra et al., 1991) |
|  | *Rytigynia decussata* (K. Schum.) Robyns (Rubiaceae) | Matapulo (Zaramo) | 1507 | Pwani | Roots | Against menorrhagia | Root decoction taken orally |  | (Chhabra et al., 1991) |
|  | *Tarenna littoralis* (Hiern) Bridson | Dungura, Mchokowejini (Zaramo), Kijiti cha buga (Swahili) | 551 1395 | Pwani | Roots | Against amenorrhoea | Root decoction taken orally | Coughs Chest pain | (Chhabra et al., 1991) |
|  | *Spermacoce dibrachiata* Oliver | Mkodakwangerekha |  | Njombe | Whole plant | For managing Menstrual disorder |  | Cataract | (Kayombo, 2016) |
|  | *Keetia venosa* (Oliv.) Bridson | Mkandachuma (Swahili) |  | DSM |  | Menstruation Miscariage |  | Sepsis rupture | (Lindh, 2015) |
|  | *Oldenlandia corymbosa* L | Iyabia, Enkaka Aloe Vera | VR nr. 6 | Kagera | Leaves, Stems | Induction of abortion | Handful of plant is chewed |  | (Nikolajsen et al., 2011; Rasch et al., 2014) |
|  | *Canthium sp.* | Omushangati, Omujuna | VR nr.8 | Kagera | Leaves | Induction of abortion | Decoction of leaves taken orally |  | (Nikolajsen et al., 2011; Rasch et al., 2014) |
|  | *Biophytum helenae* Buscal & Muschi | Webumbe | VR nr.7 | Kagera | Leaves, Stems | Induction of abortion | Leaves and stems may be chewed, boiled or drunk |  | (Nikolajsen et al., 2011) |
|  | *Rubia cordifolia* L | Akaramata | PHS nr.48 | Kagera | Leaves, Stems | Induction of abortion | Decoction of leaves and stems taken orally |  | (Nikolajsen et al., 2011; Rasch et al., 2014) |
|  | *Coffea arabica* L. | Ikawa (Kinyarwanda) |  | Kagera | Leaves | For expulsion of placenta | Infusion of the leaves taken orally |  | (Ramathal and Ngassapa, 2001) |
|  | *Oxyanthus sp.* | Mpwaga |  | Morogoro, Iringa | Roots | Infertility | A decoction from the roots is taken orally for seven days |  | (Shangali et al., 2008) |
|  | *Multidentia crassa* (Hiern) Bridson and Verdc.var crassa | Mukumbakumba, Muyogoyogo | Augustino 145 | Tabora | Roots | Infertility | Oral | Convulsion, stomach ache | (Augustino et al., 2011) |
|  | *Catunaregam spinosa* (Thunb.) Tirveng. | Mupogole, Ng’ochangoko, Ng’wiwansungu | Augustino 167 | Tabora | Roots, bark | Infertility, abortion | Oral | Gonorrhoea, hernia, stomach ache, convulsion | (Augustino et al., 2011) |
|  | *Rothmannia engleriana* (K.Schum.) Keay | Mukondokondo | Augustino 142 | Tabora | Roots, leaves, bark | Infertility, antiabortion, placenta expulsion | Oral | Gonorrhoea | (Augustino et al., 2011) |
|  | *Tapiphyllum cinerascens* (Hiern) Robyns | Kambolambola | Augustino 107 | Tabora | Roots | Infertility | Oral |  | (Augustino et al., 2011) |
|  | *Fadogia cienkowskii* Schweinf. | Kambolambola |  | Tabora | Roots | Infertility | Oral |  | (Ruffo, 1991) |
|  | *Hymenodictyon parvifolium* Oliv. | Muginya, Mujunguluji |  | Tabora | Roots | Dysmenorrhoea | Decoction; oral |  | (Ruffo, 1991) |
|  | *Chassalia violacea* K. Schum. | Mdunula |  | Iringa | Roots | Inferility |  | Infants complications, Mental case, Syphilis, Pneumonia | (Augustino and Gillah, 2005) |
| Rutaceae | *Clausena anisata*(Willd.) Hook.f. | Mjavikali (Zigua) Mkomavikali (Kwere) Mkwingwini (Pare) Mkodakwadegedege | 250 429 2029 | Pwani, Njombe | Roots | Against menorrhagia Treat irregular menses to women, As a tonic by pregnant women, Facilitates child birth and cleanses the uterus, To promote milk production, Treats threatening abortion | Root decoction taken orally, Infusion of roots taken orally | Skin diseases, Epileps, Leprosy, Syphilis, Headaches, Malaria, Influenza, Indigestiion, Anthelmintic, Diaphoretic, Rheumatic fever, Antimicrobial, Fever, Headache, Gastrointestinal disorders, Pneumonia | (Chhabra et al., 1991; Kayombo, 2016) |
|  | *Zanthoxylum chalybeum* Engl. | Mhunungu, Mjafari (Swahili, Zaramo), Mkungu, Mkiti (Zigua), Msele (Pare), Namavele (Makua), Mulungulungu, Munungu, Oluisuki | 129, 1214, 1479, Augustino 153 | Pwani, Tabora, Dodoma, Mbeya and Morogoro | Roots, Leaves, barks | Against hypermenorrhoea, Against dysmenorrhoea, Against female infertility, Women's complications, Abortion | Root decoction taken orally | Stomachaches, Strangulated hernia, Schistosomiasis, Skin disease, Swellings, Whooping cough, Aphrodisiac, and Asthma | (Augustino et al., 2011; Augustino and Gillah, 2005; Chhabra et al., 1991; Hilonga et al., 2019) |
|  | *Zanthoxylum sp* | Mjafari (Swahili) Mwale (Zigua) Oloisuki (Masai) |  | DSM |  | Right after birth |  | Anaemia STDs | (Lindh, 2015) |
|  | *Harrisonia abyssinica* Oliv | Lisawa (Jita), Mnkusu, Mkusu, Mdengwe (Sambaa) | SMM-BD10, 106 129 | Mara, Tanga | Roots | Against dysmenorrheoea | Decoction of roots taken orally | Fever, Malaria, Diarrhoea, Abscess, Fever, Insomnia, Nausea, Vomiting, Bubonic plague, Testicles swelling, Tuberculosis, Dyspepsia, Cancer, Abcesses | (Hedberg et al., 1983; Lindh, 2015; Maregesi et al., 2007a) |
|  | *Citrus sinensis* (L.) Osbeck | Omudimu, Omubirizi | PHS nr.61 | Kagera | Roots | Induction of abortion | Decoction of roots taken orally |  | (Nikolajsen et al., 2011; Rasch et al., 2014) |
|  | *Citrus limon* (L.) Osbeck |  |  | Morogoro |  | Used by breastfeeding mother as Galactagogue |  |  | (Millinga et al., 2022) |
|  | *Vepris nobilis* (Delile) W. Mziray | Njuu |  | Morogoro | Root | Dysmenorrhoea |  | Flu | (Hilonga et al., 2019) |
|  | *Toddalia asiatica* (L.) Lam. | Mtanula/ Kitanula |  | Tanga, Morogoro, Iringa | Roots | Infertility in women | Decoction; oral | Malaria, chest pain, convulsions in children, Coughs, sore throat, asthma, gonorrhea, malaria, yellow fever, toothache, intestinal worms | (Kideghesho and Msuya, 2010; Shangali et al., 2008) |
|  | *Vepris glomerata* (F. Hoffm.) Engl. | Mulungusigiti | Augustino 154, ITM 3711 | Tabora | Roots, leaves | Infertility | Oral, massage, external | Scrotal masses, aphrodisiac, hernia, diabetes, constipation, snakebite, rituals | (Augustino et al., 2011) |
| Salicaceae | *Casearia gladiiformis* Mast. | Mlelulelu |  | Iringa | Roots | Conception | Boil then drink |  | (Kitula, 2007) |
|  | *Flacourtia indica* (Burm.f.) Merr. | Musingila, Muchongoma, Mubuguswa | Augustino 176 | Tabora | Roots, leaves | Infertility | Oral, chew, massage | stomach ache, cough, hernia, snakebite | (Augustino et al., 2011) |
| Salvadoraceae | *Salvadora persica* L. | Mkayo (Pare) Mswaki (Swahili) | 3180 | Kilimanjaro | Roots | Against female sterility | Root decoction taken orally | Gonorrhea, Stomachaches, Spleen troubles, Chest diseases, Vesical catarrh, Gum disease, Gastritis, Ascarifuge and Antibacterial | (Chhabra et al., 1991) |
|  | *Allophylus africanus* Beauv | Mshongoo (Chagga) | 2984 | Kilimanjaro | Roots | Induce labour | Root decoction taken orally | Convulsions, Epilepsy, Sedative, Hernia and Cardiac pains | (Chhabra et al., 1991) |
|  | *Allophylus rubifolius* (Hochst. Ex A. Rich) Engl. | Maambakatatu, Msempelele (Zaramo) Mhecha (Ngindo) Mkongodeka (Zigua) Mwanga (Swahili) | 947 1757 | Pwani Morogoro | Roots | Against delayed pregnancy Against menorrhagia | Root decoction taken orally | Stomachaches, Fever, Mental illness, Conjunctivitis, Anemia, Constipation in children, Asthma, General swellings, Headache, Toothaches, Diarrhaea and Antibacterial | (Chhabra et al., 1991) |
|  | *Blighia unijugata* Baker | Kindamo (Ndengereko) | 1971 | Pwani | Roots, Leaves | Against Amenorrhoea | Roots and leaves decoction taken orally | Fever and Giddiness | (Chhabra et al., 1991) |
|  | *Dodonaea viscosa* (L.) Jacq. | Mjarabati (Zaramo) | 2874 | Pwani | Roots | Against irregular menstruation | Roots decoction taken orally | Indigestion, Peptic ulcers, Antipruritic and Haemorrhoids | (Chhabra et al., 1991) |
|  | *Lecaniodiscus fraxixifolius* Baker | Mbwewe (Zigua) | 1457 1498 | Pwani | Roots | Against female sterility Galactagogue | Roots decoction taken orally | Vomitting | (Chhabra et al., 1991) |
|  | *Paullinia pinnata* L. | Lugoto (Zigua) | 17 | Pwani | Roots | Against threatened abortions Expel placenta | Roots and leaves decoction taken orally | Nausea, Vomitting, Eczema, Tonic, Styptic, Snake bites, Rabies, Mental Problems, Gonorrhoea, Paralysis, Wounds, Malarian Ancylostomasis and Skin conditions | (Chhabra et al., 1991) |
| Sapindaceae | *Zanha golungensis* Hiern | Mhomavikali (Zigua) | 442 | Pwani | Roots | Against uterine prolapse Against amenorrhoea Galactagogue | Roots decoction taken orally | Hernia Chest complaints Malaria Headaches Catarrh | (Chhabra et al., 1991) |
|  | *Zanha africana* Exell | Ermerorai,Mwatia, Mdaula | 242 | Dodoma, Mbeya and Morogoro | Root bark | Facilitate child birth, Dysmenorrhoea | Decoction taken orally | Fungal infections, Aching legs, Constipation, Prostatitis Fits, migraine, cough, Libido disorder | (Chhabra et al., 1984; Hilonga et al., 2019) |
|  | *Allophylus rubifolius*  (Hochst. ex A. Rich.) Engl. | Msempele (Zigua) | 222 | Tanga | Roots | To make birth easy | Decoction of roots taken orally | Diarrhoea mouthwash Toothache | (Hedberg et al., 1983) |
|  | *Deinbollia borbonica* | Mmoyomoyo |  | Tanga | Roots | Infertility to women | Decoction; oral | Hernia | (Kideghesho and Msuya, 2010) |
| Sapotaceae | *Pachystela msolo* (Engl.) Engl. | Msambia (Pale) | 291 | Kilimanjaro | Stembark | Lactagogue | Decoction of stem bark and sugar cane taken orally |  | (Hedberg et al., 1983) |
| Sterculiaceae | *Dombeya cfr. cincinnata* K. Schum. | Mkwelengala (Zigua) | 200 | Tanga | Roots | Against dysmenorrhoea | Decoction of roots mixed with Mkusu and Mrundwi taken orally |  | (Hedberg et al., 1983) |
|  | *Dombeya shupangae* K. Schum. | Mnwati (Pare) Mkiika (Sambaa) Muwati (Sambaa) | 255 287 | Kilimanjaro | Roots | Against amenorrhoea Prevent abortion(leaves) | Decoction of roots taken orally Infusion of leaves | Stomach pain, Constipation, and Wound healing | (Hedberg et al., 1983) |
|  | *Sterculia cfr. stenocarpa* H. Winkler | Mhoza (Moza) | 133 | Tanga | Stembark | Cures abdominal pains accompanied by mild bleeding from the vagina. | Decoction of the fresh stembark and fresh bark of Mfune, Mbuyu, and Shiizi |  | (Hedberg et al., 1983) |
|  | *Waltheria indica* L. |  | 74 | Tanga | Whole plant | Abortifacient Against barrenness |  | Wound, Convulsions, Syphilis, Cough, Astringent, Eye ache, Internal hemorrhage, Purgatives, Fever, Dysentry, Epilepsy and Syphilis | (Hedberg et al., 1983) |
|  | *Sterculia quinqueloba* | Mparamisi (mzungupori) jike | RMK007 | Kigoma | Leaves | Dilating cervix to aid easy delivery/reducing dryness. | Paste prepared from clean fresh leaves is mix with palm oil (or other vegetable oil) is applied through the vagina. |  | (Kingo and Maregesi, 2020) |
| Strychnaceae | *Strychnos innocua* Delile | Mumundu | Augustino 157 | Tabora | Roots | Infertility | Oral | Aphrodisiac | (Augustino et al., 2011) |
|  | *Strychnos spinosa* Lam. | Mwage | Augustino 195, ITM 3704 | Tabora | Roots, leaves, bark | Infertility | Oral, chew, massage | Tumours, convulsion, vomiting, intestinal worms, stabbing sensations, gonorrhoea, syphilis, cough, stomach ache, snakebite | (Augustino et al., 2011) |
| Tiliaceae | *Triumfetta brachyceras* Syn; Triumfetta macrophylla Wight & Arn. | Kikikarabo | PHS nr.53 | Kagera | Leaves, Stems Flowers | Induction of abortion | Infusion of the plant taken orally |  | (Nikolajsen et al., 2011) |
|  | *Grewia bicolor* Juss | Olesiteti |  | Iringa | Roots | Infertility, Missing menstrual cycle |  |  | (Augustino and Gillah, 2005) |
| Urticaceae | *Obetia radula* (Baker) B.D.Jacks | Ekijumbula | PHS nr.50 | Kagera | Leaves, Stems | Induction of abortion | Decoction of leaves and stems taken orally |  | (Nikolajsen et al., 2011; Rasch et al., 2014) |
| Verbenaceae | *Lantana viburnoides* (Forsk.) Vahl. | Mvuti (Sambaa) | 175 | Tanga | Roots | Used when a woman cannot be pregnant. | A decoction of the roots with the roots of Mhasha taken orally |  | (Hedberg et al., 1983) |
| Vitaceae | *Rhoicissus revoilii* Planch (Ampelidaceae) | Funga ng'ombe, Mfungangombe (Zigua), Ilyungulyungu | 106 243 | Tanga, Pwani | Rootbark, roots | Against menstrual disorders, Avoid abortion, Infertility | Decoction of root bark taken orally | Cuts, Wounds, Sores, Burns, Anaesthetic | (Abdallah et al., 2007; Chhabra et al., 1984; Hedberg et al., 1983) |
|  | *Rhoicissus tridentata* (L.F) Willd et Drum. | Mgomogomo | 2986 |  | Leave, Roots | Against excessive menses Cure abdominal pain during menstruation | Decoction of leaves taken orally Decoction of roots taken orally | Indigestion Sores and Cuts Anaesthesia | (Chhabra et al., 1984) |
|  | *Cyphostemma adenocaule* (Steud. ex A. Rich.) Descoings | Mwengere, Mwangele (Kimakonde) | 16 154 | Tanga | Roots | To reduce excessive menstrual bleeding. Treat abdominal pain during pregnancy Prevent abortion | A decoction of the roots with Mserere, Kivunjayuki and Mdimu taken orally | Stomach pain, Migraine, Mental diseases, Wounds Joint disease, Swellings, Syphilis and Purgative | (Hedberg et al., 1983) |
|  | *Cyphostemma sp.* | Mwengele (Zigua/Swahili) |  | DSM |  | Infertility Pregnancy |  |  | (Lindh, 2015) |
|  | *Cissus rotundifolia* Lam. | Mkilua |  | Pwani | Roots | Infertility; Menorrhagia | Decoction; oral |  | (Abdallah et al., 2007) |
|  | *Cyphostemma paucidentatum* (Klotzsch) Desc. ex Wild & R.B.Drumm. | Mwengele |  | Pwani | Roots | Infertility; Menorrhagia | Decoction; oral. Infusion; oral |  | (Abdallah et al., 2007) |
| Zingiberaceae | *Zingiber officinale* Roscoe |  |  | Mwanza, Morogoro |  | labor induction, Used by breastfeeding mother as Galactagogue |  |  | (Dika et al., 2017; Millinga et al., 2022) |
| Zygophyllaceae | *Balanites aegyptiaca* (L.) Delile | Olng’oswai | Augustino 208 | Tabora | Roots | Menstrual disorders | Oral |  | (Augustino et al., 2011) |
